# Biogeography of the coastal fishes of the Socotra Archipelago: Challenging current ecoregional concepts

**DOI:** 10.1371/journal.pone.0267086

**Published:** 2022-04-29

**Authors:** Uwe Zajonz, Edouard Lavergne, Sergey V. Bogorodsky, Friedhelm Krupp

**Affiliations:** 1 Ichthyology Section, Department of Marine Zoology, Senckenberg Research Institute and Museum of Nature, Senckenberg Society of Nature Research, Frankfurt am Main, Germany; 2 Department of Biogeography and Ecosystem Research, Quantitative Biogeography Group, Senckenberg Biodiversity and Climate Research Centre (SBiK-F), Senckenberg Society of Nature Research, Frankfurt am Main, Germany; 3 Educational Unit for Studies on Connectivity of Hills, Humans and Oceans (CoHHO), Field Science Education and Research Center (FSERC), Kyoto University, Kyoto, Japan; MARE – Marine and Environmental Sciences Centre, PORTUGAL

## Abstract

The Socotra Archipelago, located in the eastern Gulf of Aden, has a unique marine environment, which combines tropical and ‘pseudo-temperate’ elements. An updated species inventory recently considered its coastal fish diversity the highest among Arabian ecoregions, necessitating to re-assess the ichthyogeographic position of the island group. The main aim of this study is to describe the distributional biogeography of its coastal fish fauna in relation to contemporary ichthyogeographic and ecoregional concepts. Inferences are drawn with regard to the marine biogeographic arrangement and ecoregional partitioning of the Arabian region. The main datasets comprise eight and twenty selected families including 404 and 898 species, respectively, from Arabian ecoregions. The Socotra Archipelago has close affinities to a putative ecoregion in the eastern Gulf of Aden that extends to southern Oman. It is more closely related to the Arabian Sea coast of Oman than to ecoregions in the Red Sea and a putative ecoregion in the western Gulf of Aden. The Gulf of Aden does not represent a consistent ecoregion in ichthyogeographic terms, because its eastern and western parts are less closely related to one another than to other ecoregions. The Socotra Archipelago and the eastern Gulf of Aden should therefore not be assigned to a joined province with Red Sea ecoregions. The coastal fish faunas of the southern Red Sea have close affinities with those of the western Gulf of Aden. The Arabian/Persian Gulf is least related to the other Arabian ecoregions. The authors posit the Socotra Archipelago as a distinct ecoregion, either on its own or in combination with affiliated mainland areas. This best reflects the ichthyogeographic data and the exceptionally high levels of fish and overall marine diversity. Two alternative ecoregional delineations are proposed, serving as working hypotheses for onward research.

## Introduction

The Socotra Archipelago (Yemen) in the northern Indian Ocean is recognized globally for its outstanding universal values, including unique patterns of biodiversity, which led to the designation of the entire island group as a UNESCO World Heritage Site in 2008 [1–3]. The Archipelago lies at the centre of a region with inadequately documented fish faunas [4]. Kemp (1998) [5] was the first to assess coastal fish assemblages of the island group, after the historic studies by Steindachner (1902, 1903) [6, 7]. He reported 215 species based on visual records and provided an initial regional zoogeographical analysis (compare also Kemp 2000) [8]. Subsequent surveys [9, 10] revealed that the fish species richness is substantially higher. It includes unique coral-associated fish assemblages, in spite of scarce biogenic reef frameworks [11, 12]. A study by DiBattista et al. (2015) [13] underestimated species richness values (514-577 species) of Socotra. A recent preliminary checklist by Zajonz et al. (2019) [4] includes 733 species in 108 families. Extrapolated fish species richness was 875, exceeding values in adjacent Arabian ecoregions. The study also reviewed the history of ichthyological research in the Socotra Archipelago.

The study area and its biogeographically relevant environmental variables were described by e.g. [12, 14, 15]. Information on fish community ecology can be found in [4, 15, 16].

The marine biogeographic affinities of the Archipelago were summarised by e.g., [5, 11, 12, 17–19]. DeVantier et al. (2004) [11] characterised the particular biogeographic attributes of the Archipelago as a “zoogeographic crossroads”.

It has long been recognized that, ichthyogeographically, the Socotra Archipelago is located at the intersection of several distinct biogeographic units, based on studies by e.g., [20–27]. The hypothetical ichthyogeographic boundaries and putative barriers that cause faunal breaks in the seas around the Archipelago are still a matter of scientific debate (e.g., [13, 28–35], as will be reflected in the discussion. The study by DiBattista et al. (2015) [29] is especially noteworthy because it recognized the Archipelago as a globally outstanding hotspot of marine fish hybridisation. Hybridization per se is not investigated in the present study yet briefly discussed.

Zajonz et al. (2019) [4] classified a total of 658 species according to 12 main categories of distribution range patterns, and reviewed the biogeographic literature on the Socotra Archipelago and surrounding regions, recognizing substantial recent advances in phylogeographical studies of the Arabian Region, while identifying critical knowledge gaps, particularly regarding distributional and ecological biogeography in the wider Gulf of Aden–Socotra–Somalia sector. The results of both, recent global and regional studies were considered not to be congruent in a satisfactory way, and based on outdated species lists of the Socotra Archipelago and southern Arabia. The present article is concerned with the following issues:

Kemp (1998) [5] based his study primarily on the regional distribution of five families of reef-associated fishes, with a focus on Chaetodontidae. In this study and three subsequent articles ([8, 36, 37]) he advanced the knowledge of the marine biogeography of the north-western Indian Ocean substantially. He suggested a “South Arabian region”, combining parts of southern Oman and eastern Yemen, and recognised strong affinities of this region with the Socotra Archipelago, next to a parallel “East African influence”. Since Kemp’s surveys the number of species recorded has more than tripled, making it necessary to update the biogeographic analyses. Moreover, Kemp’s studies imply that the Gulf of Aden is probably not a homogenous biogeographic unit; an important point that has been largely ignored in the subsequent literature.

The global studies of Spalding et al. (2007, Marine Ecoregions of the World, MEOW) [38], Briggs and Bowen (2012) [39] and Kulbicki et al. (2013) [40] included inferences about the potential marine ecoregional and biogeographic position of the Archipelago and the delineation of the southern Arabian region. In terms of fish distribution data, they were based on limited faunal records, i.e. those of Kemp (1998) [5].

**Fig 1:**
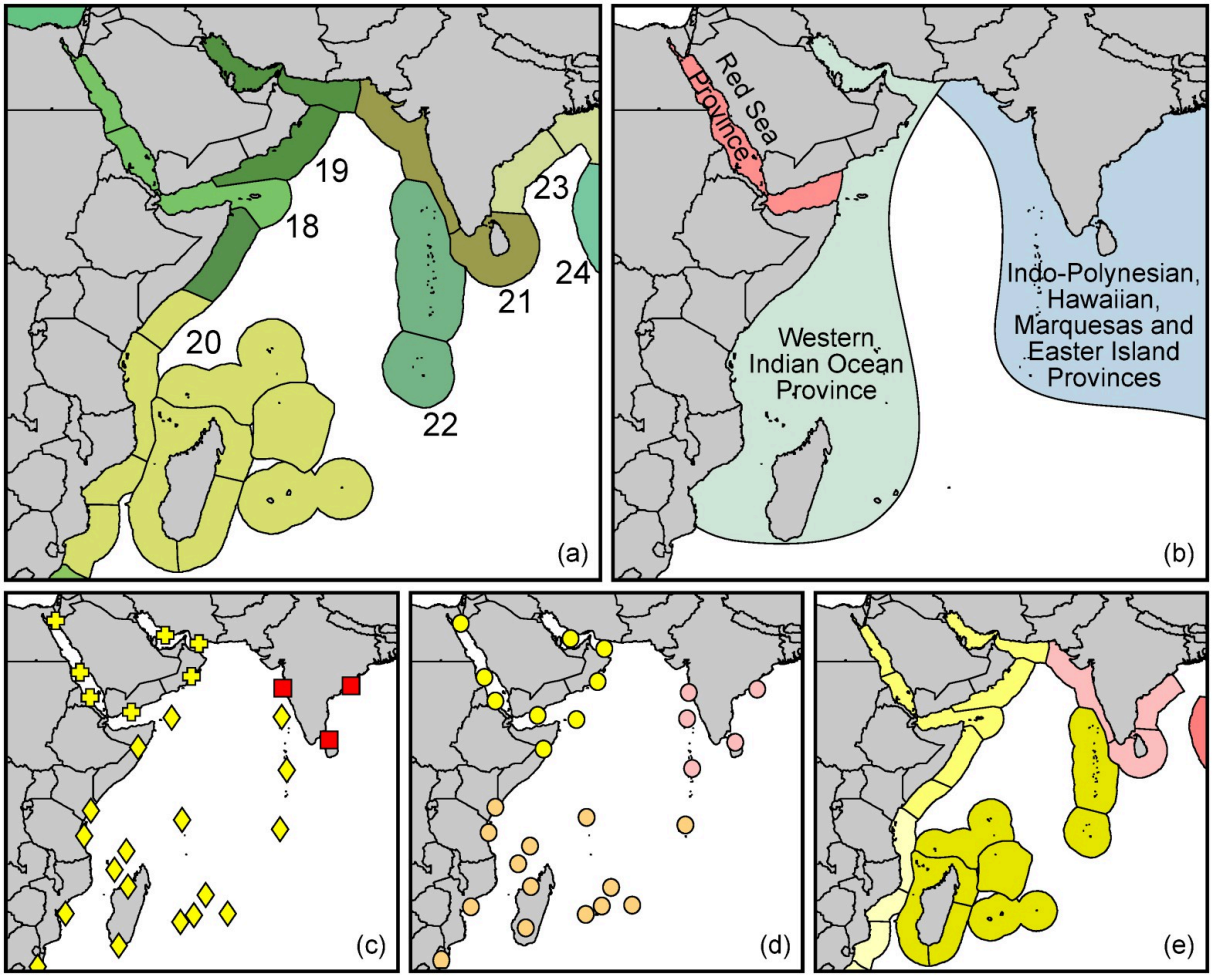
North-western Indian Ocean sections of three current global marine “biogeographic” classification schemes: (a) ‘Marine Ecoregions’ (Spalding et al. 2007), (b) ‘Marine/Fish Biogeography’ (Briggs and Bowen 2012), (c-e) ‘Global Reef Fish Biogeography’ (Kulbicki et al. 2013, with three alternatives); as evaluated in the present article. (a-e: Baseline map sourced 2019 from Natural Earth, free vector and raster map data <naturalearthdata.com> with no copyright restrictions. a, e: Ecoregion boundaries redrawn from GIS data accompanying Spalding et al. (2007), sourced 2012 from <conserveonline.org>, with free permission for scientific use and reproduction (to date only available from <worldwildlife.org>), colours modified.b-d: Redrawn manually by the authors according to the original publications, colours modified).

Spalding et al. (2007) [38] presented a global hierarchical bioregional classification system (Fig 1a) composed of 12 realms, 62 provinces, and 232 ecoregions. The Socotra Archipelago was assigned to the ‘Gulf of Aden’ ecoregion (E89; following the enumeration of Spalding et al., adding for clarity the capital letters ‘E’ for ecoregion-level and ‘P’ for province-level numbering), part of the ‘Red Sea–Gulf of Aden’ province (P18), within the ‘Western Indo-Pacific’ realm. The MEOW scheme has been widely adopted since, partly with modifications though, e.g., [13, 41]. Ecoregions were defined by Spalding et al. (2007) [38] as “ecologically and taxonomically homogeneous and strongly cohesive units”. Whether, however, these prerequisites apply to the ecoregion Gulf of Aden, including the Socotra Archipelago, is challenged in the present study, at least with regards to ichthyogeographic data.

Updating earlier marine biogeographic concepts (i.e. [22, 42, 43]) Briggs and Bowen (2012) [39] proposed a realignment of marine biogeographic provinces with special regard to fish distributions (Fig 1b). According to these authors, the Socotra Archipelago belongs to a ‘Western Indian Ocean Province’—within a ‘Tropical Indo-West-Pacific Region’—that extends along the East African coast from south of Madagascar northwards to the Gulf of Oman, and also includes the Arabian/Persian Gulf. With regard to the larger Gulf of Aden they sharply delineate this province to the west, along a line from the Horn of Africa to Ras Fartak in eastern Yemen. They combined the inner Gulf of Aden with the Red Sea proper into a ‘Red Sea’ province, excluding the Socotra Archipelago. If compared to the MEOW of [38], several important aspects require further attention: (a) Briggs and Bowen’s delineation of the Gulf of Aden differs from MEOW in that the Socotra Archipelago belongs to a different biogeographic unit than the western part of the Gulf of Aden; therefore (b) it separates the Archipelago from the Red Sea at the provincial level, unlike in MEOW. The Western Indian Ocean province of Briggs and Bowen (2012) [39] encompasses two provinces of MEOW [38]. Briggs and Bowen (2012) [39] accordingly do not recognize a single, cohesive circum-Arabian biogeographical unit, while the MEOW would allow for it to a certain extent. Obura (2012) [41], while applying the MEOW scheme, proposed a “pan-Arabian province” based on coral assemblage data. A hypothetical “pan-Arabian fish province” should be put to the test.

Kulbicki et al. (2013) [40] provided a global biogeography of tropical reef fishes (Fig 1c–1e), based on a hierarchical, quantitative delineation of biogeographic units derived from a global database composed of 169 checklists (including 163 lists compiled by Parravicini et al. 2013 [44]). Relying on the outdated checklist of Kemp (1998) [5], they were not able to resolve the position of the Archipelago, placing it along with “Somalia” in either a ‘Western Indian Ocean’ province based on all species or a ‘North-western Indian Ocean’ province based on species they consider “reliable” (see Fig 4a, 4b of [40]). In a second methodological approach they assigned their checklist data *a priori* to MEOW. Because of the data assignment to predefined spatial units no specific biogeographic signal was detected for the Socotra Archipelago. The resulting higher level biogeographic units, ‘provinces’ and ‘realms’, neither conform to their aforementioned results, nor to MEOW [38], nor to Briggs and Bowen (2012) [39].

The main aim of this article is to characterize the distributional biogeography of the coastal fishes of the Socotra Archipelago in the context of contemporary biogeographic and ecoregional concepts, with a focus on the ‘Marine Ecoregions of the World’ of Spalding et al. (2007) [38]. The working hypotheses include: (i) the wider Gulf of Aden does not represent a consistent ecoregion in terms of fish assemblage composition; (ii) the eastern Gulf of Aden is more closely related to Socotra and to southern Oman than to its western part, and southern Oman is more closely related to the former than to central Oman; and, (iii) the Socotra Archipelago does represent an ecoregion of its own, optionally with affiliated mainland areas, and does not share a province-level unit with Red Sea ecoregions.

## Materials and methods

**Fig 2:**
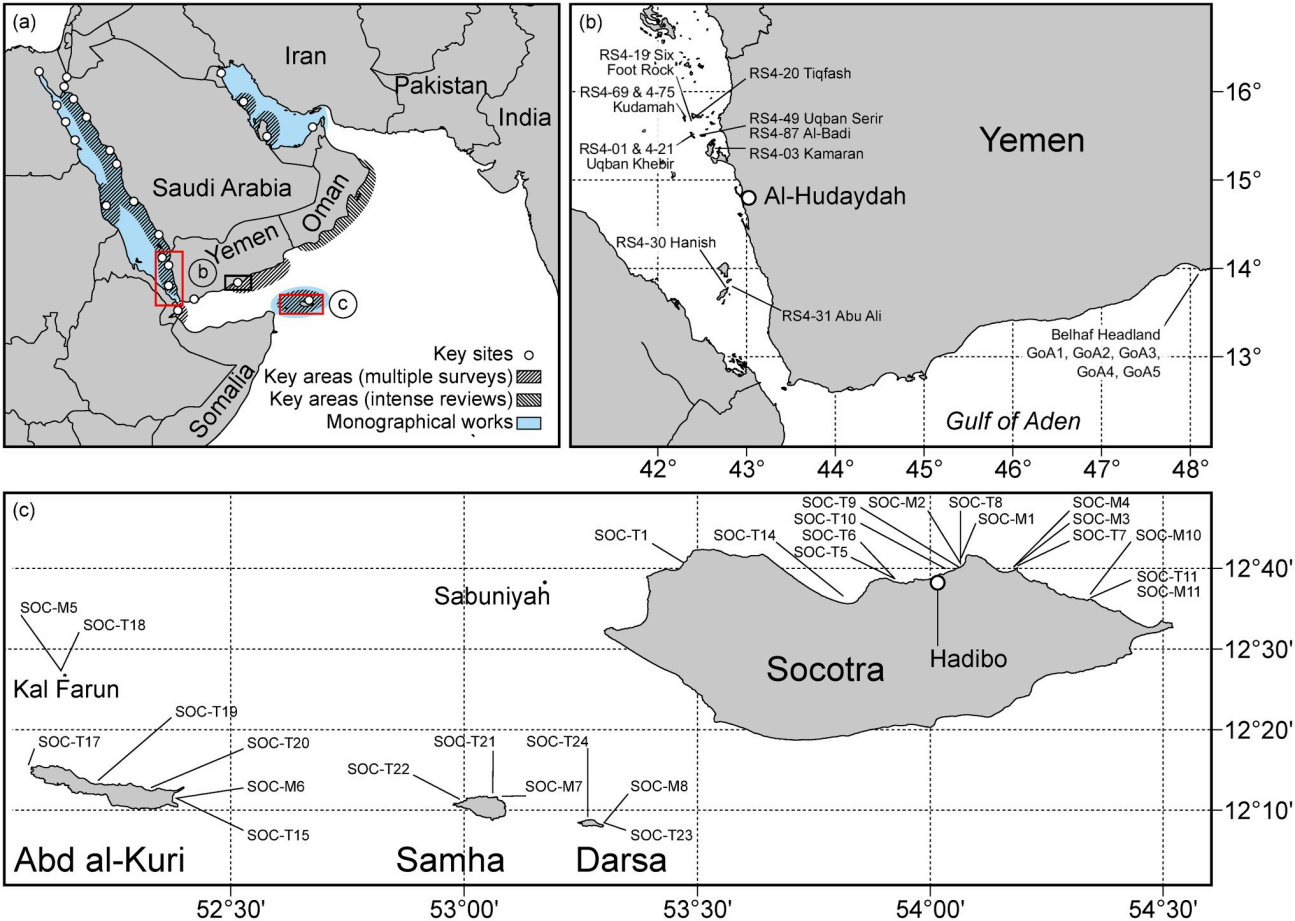
Overview maps of the study area, showing a) areas in the Arabian Region studied by the authors, including the geographic location of the Socotra Archipelago in the north-western Indian Ocean and the main survey areas included in the subregional analyses (rectangles), with b) fish inventory survey sites including four transect sites in the Yemeni Red Sea of 2003 and 2004, and c) transect sites (T) and ecological monitoring sites (M) in the Socotra Archipelago from 1999 to 2007. The transect sites at Ras Belhaf at the mainland Gulf of Aden coast of Yemen of 2005 are not shown on a separate map. (a: Baseline map sourced 2019 from Natural Earth, free vector and raster map data <naturalearthdata.com> with no copyright restrictions.b: Baseline map sourced 2019 from <commons.wikimedia.org/wiki/File:Yemen_location_map.svg> under license <CC-BY-SA-3.0> unported license, made or improved in the German <Kartenwerkstatt> (Map Lab).c: Baseline map owned by the authors, sourced from GIS data based on LandSat TM5 satellite images owned by the authors (map credits: R. Klaus)).

The present study draws primarily on data collected by the authors during more than 40 years of field research throughout the Arabian Region (Fig 2), including the author’s own publications and unpublished distributional databases.

It mainly analyses incidence-based (presence-absence and presence) data of eight key families: Acanthuridae, Balistidae, Chaetodontidae, Pomacanthidae, Pomacentridae, Labridae, Pseudochromidae, and Serranidae, with 404 species from Arabia s.lat. including 234 species from Socotra. These families were chosen following Zajonz et al. (2019) [4] because they represent a combination of taxa which are biologically diverse (in terms of trophic, functional, reproductive and dispersal traits), biogeographically informative (sufficiently unevenly distributed while including few zeros at the family level), taxonomically well studied and reliably identified to species level, both in the field and in the laboratory. The first five families were also selected to ensure comparability with Kemp (1998, 2000) [5, 8] whose works, focussing on those families, form a major backdrop to the present study.

In order to provide additional evidence complementary analyses of larger data sets are conducted, involving all recorded families, pertinent to the subregional analyses, and twelve additional core families, pertinent to the regional analyses. The latter of which includes the Apogonidae, Blenniidae, Carangidae, Haemulidae, Holocentridae, Lethrinidae, Lutjanidae, Mullidae, Muraenidae, Scaridae, Scorpaenidae, and Tetraodontidae. In combination with the initial eight key families this regional data set comprises twenty families, with 898 species from Arabia *s*.*lat*. including 463 species from Socotra, covering well more than half of all species known from both areas.

First, the resemblance of the Socotra Archipelago’s coastal fish assemblages is analysed at the ‘subregional’ spatial scale (encompassing the Socotra Archipelago, northern Gulf of Aden and southern Red Sea), based on the authors’ presence-absence data. Second, the assemblages are investigated at the ‘regional’ scale (sea area surrounding the Arabian Peninsula), based on presence data (faunal lists). The latter were compiled from the authors’ data, published records and “grey” sources (including FishBase [45]) that were critically reviewed according to the latest literature and the authors’ expertise.

The qualitative (presence-absence) and quantitative (abundance) data from the Socotra Archipelago (Fig 2c) and the additional subregional sites (Fig 2b) were primarily collected between 1996 and 2014. Faunistic records were added until 2019. Field survey and sampling methods, and taxonomic literature used are given in Zajonz et al. (2019) [4]. Comprehensive descriptions of key methods are found in [5, 9, 10, 16, 46], and additional information is found in [15]. Pertinent to the subregional analyses, the data packages are semi-standardized in that records of underwater belt-transects (based on [47]) were combined with records of detailed fish inventory sites (see [4]) of comparable survey effort (both using SCUBA), including surveys of different years. This addressed the data-poor context and the difficulties to survey those areas. Accordingly, abundance data were not used and ‘Hellinger Distance’ was chosen because it represents a resemblance index that is robust against not fully standardised sampling efforts [48]. Faunistic data for Socotra Archipelago used in the regional analyses are from the preliminary checklist in Annex 1 of Zajonz et al. (2019) [4], spanning more than 20 years of field recording.

All multivariate analyses were conducted using the ecostatistic software ‘Plymouth Routines in Multivariate Ecological Research’ (PRIMER v6) following [49, 50]. The PRIMER package assumes that hypotheses of normality [51] and homoscedasticity [52] are not met for marine multivariate datasets. For the resemblance analyses pairwise dis-/similarity matrices were calculated from binary input matrices of presence-absence or presence data, based on Hellinger distance (samples or aggregated areas” as columns, by species as rows). The procedure was repeated for validation using the similarity indices ‘Jaccard’, ‘Ochiai’ [53] and ‘Bray-Curtis’ [54], with the latter being equivalent to the ‘Soerensen’ index when applied to incidence data [49].

The ‘Analysis of Similarities’ routine ANOSIM [49, 55] permits testing for differences in multivariate data structures between *a priori* defined groups. ANOSIM was used to test the validity (Global R) and significance (p) of hypothetical spatial structures at levels higher than that of the data fed into the resemblance analyses. The hypothetical spatial designations were defined as options for one or several ‘factors’ in the PRIMER input matrix. The samples (columns) were *a priori* classified accordingly, i.e. before the results of the resemblance analyses were available, in order for the tests to be statistically correct [49].

Hierarchical Agglomerative Cluster Analysis was applied to further explore the relatedness of the samples beyond any spatial structure vested in the data compilation or implied by the biogeographic schemes tested, according to the pairwise dis-/similarities using Group Average Linkage (e.g. [56]). The resulting combinations were plotted into cluster dendrograms for visual examination. Non-metric Multi-dimensional Scaling (nMDS) was used as an alternative ordination method, i.e. to plot spatial structures supported by ANOSIM (e.g., [55, 57, 58], with 100 iterations, Kruskal Stress formula 1, and 0.01 minimum stress level as standard settings. The ‘Similarity Profile Analysis’ routine SIMPROF was conducted to test for structure in the data and statistically validate the relatedness of the cluster branches, with 2000 permutations used to generate the mean profile and 999 simulations to calculate the statistics, and 5% significance level as standard settings. Insignificant relations are illustrated by red branches in the dendrograms. The Ochiai similarity consistently produced closely concordant results compared to those obtained with the Hellinger distance, both in terms of resemblance patterns and SIMPROF significance of data structure. The Jaccard and Bray-Curtis/Soerensen based results usually conformed in terms of resemblance patterns, yet often produced lower SIMPROF support to terminal branches.

The ‘Similarity Percentages’ routine SIMPER complemented the regional analyses by assessing the statistically most valid *a priori*-defined ‘province-level combination’ of ecoregions identified by the ANOSIM. The procedure firstly calculates the average within-similarity of all groups of samples based on the Bray-Curtis index, secondly the average dissimilarity between all pairs of groups of samples, and thirdly, the average percentage contribution of each species to the average gross dis/-similarities [49]. A cut at 75% cumulative dis/-similarities was applied, excluding species with minor individual contributions.

(S1–S4 Data, S1–S4 Figs) is presented online, as listed at the end of this article.

### Subregional resemblance patterns

For the ‘subregional’ analyses 29 samples (22—abundance-based, presence-absence transformed—fish transects and seven additional—incidence-based—fish inventory sites) were investigated. The samples comprised five and eight transects of 2003 and 2007 respectively from standard monitoring sites at Socotra Archipelago [46], whereby five samples were taken at identical sites at the main island. This combination permits to tentatively explore whether the assemblages from Socotra form a stable cluster through several years and monsoon seasons (compare [15]). The samples also included five transects of 2005 from Belhaf headland (Yemen mainland, Shabwa governorate, Gulf of Aden) and four transects and seven fish inventories of 2003 and 2004 from Hanish Archipelago and Kamaran Archipelago in the Yemeni Red Sea (Fig 2). Due to the severe logistic and security constraints in the area it had not been possible to sample those sites within a single year or to apply a consistently structured sampling scheme across several years. Though remaining explorative of necessity, the present subregional comparison is the only one to date based on consistent data by a single surveyor (U. Zajonz). Resemblance matrices based on the Hellinger distance were analysed, validated by three complementary indices, using the full data of all 29 samples (403 species), a data subset reduced to the eight key families (193 species) of interest in the present study, and a data subset reduced to the four families (65 species) primarily studied by Kemp (1998) [5] and Kemp (2000) [8]. ANOSIM test was computed according to the *a priori* factor designation ‘basin’ (Northern Indian Ocean, Gulf of Aden, and southern Red Sea), using 99999 permutations in order to verify that those geographical units are eco-statistically distinct.

### Regional resemblance patterns and alternative ecoregional arrangements

In order to characterize the biogeographic affinities of the Socotra Archipelago’s coastal fish assemblages, the resemblance of distributional data of the eight key families was analysed according to 10 putative “Arabian” marine ecoregions. The definitions and enumeration of the ecoregions were based on MEOW (Fig 1a), covering ecoregions E87-93 within the provinces Red Sea–Gulf of Aden (P18) and Somalia-Arabia (P19); but modified as follows: In altering the classification of MEOW, ecoregion E87 (Northern and Central Red Sea) was disaggregated by collating a separate species list for the Gulf of Aqaba. The ecoregion E89 (Gulf of Aden) was disaggregated by collating separate species lists for the western Gulf of Aden (strongly representing Djibouti because of data availability), the eastern Gulf of Aden (strongly representing the Shabwa, Hadhramout and Al-Mahara coasts of Yemen because of data availability), and the Socotra Archipelago (S1 Data). In contrast to the MEOW, the boundaries of the ecoregions Western Arabian Sea (E92) and Gulf of Aden (E89) were detached, as follows: The MEOW assigns most of the north-eastern Gulf of Aden and southern Oman to ecoregion E92. This does not seem to concur with Kemp [5, 8], who identified strong biogeographic affinities between Socotra Archipelago and these areas, whereas the MEOW assigns both to different provinces. Also Randall and Hoover [59] observed distinct fish assemblages in southern Oman compared to central Oman and the Gulf of Oman. Therefore, a hypothetical ecoregion was added inbetween ecoregions E92 and E89 covering the eastern Gulf of Aden and southern Oman (the Dhofar coast and Hallaniyat Islands), and also the Socotra Archipelago was added putatively as independent separate ecoregion.

In order to distinguish the enumeration of the modified putative spatial units from the original MEOW units the suffix ‘E’ for ecoregions is changed to ‘e’, and ‘P’ for provinces (~province-level) to ‘p’. Lower case letters are added behind the numbers if units are split or added, e.g. from ecoregion E89 to e89a and e89b, or from province P19 to p19a and p19b, while the MEOW numbers are kept in order to ease the cross-referencing between the original MEOW and the modified scheme.

Resulting from the above, the hypothetical ecoregional units were: e89c, Socotra Archipelago (Soc) / e87a, Gulf of Aqaba (GoAq) / e87b, Northern and Central Red Sea (NC RS) / e88, Southern Red Sea (S RS, identical with E88) / e89a, Western Gulf of Aden-Djibouti (W GoA (Djib)) / e89b, Eastern Gulf of Aden extended (by southern Oman, E GoA ext.) / e92, Central Oman (CO, remaining partly identical with northern section of the Western Arabian Coast E92) / e91, Gulf of Oman (GoO, identical with E91) / e90, Arabian/Persian Gulf (AG, identical with E90) / e93 “Somali Current Coast” (SCC, representing a surrogate for the Central Somali Coast E93 and the northern part of the Northern Monsoon Current Coast E94 of the MEOW).

The purpose of disaggregating ecoregion E89 (Gulf of Aden) was to explicitly test the homogeneity of this area (as presumed by MEOW and others), verifying the working hypothesis (i) that the wider Gulf of Aden does not represent a consistent ecoregion in terms fish assemblage composition. The separate checklist for the Socotra Archipelago was to test the working hypothesis (iii) that it represents an ecoregion of its own. Separating the north-eastern part of the Gulf of Aden and southern Oman from MEOW’s Western Arabian Coast (E92) and assigning them to a putative extended eastern Gulf of Aden (e89b) permitted to test explicitly the affinities of the Socotra Archipelago to those areas, in comparison to the remaining Arabian Sea coast of Oman (central Oman), verifying–in part–working hypothesis (ii). The checklists for the eastern Gulf of Aden and southern Oman were initially merged because the ANOSIM could only be conducted once due to its requirement to test only *a priori* assumptions of spatial data structure. A complementary resemblance analysis based on distinct checklists for both areas was conducted in order to verify the working hypothesis (ii), namely that they are closer to one another than to other ecoregions. Fish records from southern Oman are still limited and largely originate from Randall (1995) [60]. The separate species account for the Gulf of Aqaba (as e87a) was included, because this basin represents an extreme, semi-enclosed environment within the Red Sea (e.g., [13, 61]). A fish species list for Kenya (representing a surrogate for the southern part of Northern Monsoon Current Coast E94) was compiled (primarily sourced from [45, 62] and critically reviewed). It was added in order to root the analyses of the Arabian ecoregions (pertinent to the provinces P18-19) towards an adjacent province (Western Indian Ocean, P20). The resulting checklists comprise 404 species for the data set of eight key families and 898 species for the complementary data set of twenty families (S1 Data).

Resemblance matrices were calculated based on the Hellinger distance and results were compared with those of the three complementary indices for cross-validation. Dendrogram plots of the different cluster analyses (eight-families input without and with separate checklist for southern Oman; twenty-families input) were compared to deduce inferences on the plausibility of the working hypotheses.

ANOSIM was conducted, independently of the cluster analyses, to investigate the ordering of the putative ecoregional units into up-scale putative province-level units, in scrutinizing existing concepts, and to test–in part–the working hypothesis (iii) that Socotra does not share a province-level unit with Red Sea ecoregions. The term ‘province-level’ is used here provisionally to name the next higher spatial aggregation unit after ecoregions without adopting the definition of ‘province’ from any of the biogeographic schemes under scrutiny. A total of 28 plausible hypothetical *a priori* designations (combinations) of the 10 putative Arabian ecoregional units plus Kenya into province-level units were tested as ‘factors’ by ANOSIM, using 99999 permutations (Table 1, S2 Data). The *a priori* designations included (a) the proposed provinces P18 and P19 of MEOW [38]; (b) two variations of the proposed provinces ‘Red Sea’ and ‘Western Indian Ocean’ of Briggs and Bowen (2012) [39]; (c) two of the three alternative province-level classifications of Kulbicki et al. (2013) [40]; and, (d) 23 alternative combinations. The relatedness of the fish faunal composition of the putative ecoregions explored by the ordination analyses was compared to the ANOSIM results. A 29^th^ combination, representing one of the three alternative province-level classifications of Kulbicki et al. ([40]; Fig 4c, 4d) could not be tested *a priori* with ANOSIM in the present study lay-out because it assigns all presumed ecoregions into a single province, thus leaving the scale of the present study.

**Table 1:** List of *a priori*-defined ‘province-level combinations’ of putative Arabian ecoregions investigated with ANOSIM (based on a resemblance matrix calculated from distributional data of eight key families with Hellinger distance), and Global R and significance results; arranged in order of Global R (including Kenya as comparative outgroup; ‘a’ and ‘b’ coded numbers are computationally separate province-level designations).

	Putative Ecoregions ^1^												
Tested Province-level Combinations ^2^	Soc (e89c)	GoAq (e87a)	NC RS (e87b)	S RS (e88)	W GoA (e89a)	E GoA ext. (e89b)	CO(e92)	GoO (e91)	AG (e90)	SCC (e93)	Kenya	Global R (ANOSIM)	Significance %
U. ‘Arabia dividua 4b’	19a	18	18	18	18	19a	19a	19b	19b	20	20	0.975	0.003
Z. ‘Arabia dividua 5’	20	18	18	18	18	19a	19a	19b	19b	21	21	0.957	0.009
W. ‘Arabia dividua 4d’	20a	18	18	18	18	19a	19a	19b	19b	20a	20b	0.932	0.020
T. ‘Arabia dividua 4a’	19a	18	18	18	18	19a	19a	19b	19b	19a	20	0.886	0.006
V. ‘Arabia dividua 4c’	20	18	18	18	18	19a	19a	19b	19b	20	20	0.847	0.009
S. ‘Arabia dividua 3d’	20a	18	18	18	18	19a	19a	19a	19b	20a	20b	0.796	0.030
Q. ‘Arabia dividua 3b’	19a	18	18	18	18	19a	19a	19a	19b	20	20	0.788	0.010
G. ‘Arabia minima 1d’	19	18a	18a	18a	18a	18a	18a	18b	18b	19	19	0.751	0.060
R. ‘Arabia dividua 3c’	20	18	18	18	18	19a	19a	19a	19b	20	20	0.740	0.020
A1. ‘Spalding et al.’	18	18	18	18	18	18	19	19	19	19	20	0.703	0.040
O. ‘Arabia dividua 2d’	20a	18	18	18	18	19	19	19	19	20a	20b	0.674	0.090
P. ‘Arabia dividua 3a’	19a	18	18	18	18	19a	19a	19a	19b	19a	20	0.670	0.090
N. ‘Arabia dividua 2c’	20	18	18	18	18	19	19	19	19	20	20	0.653	0.030
M. ‘Arabia dividua 2b’	19	18	18	18	18	19	19	19	19	20	20	0.622	0.100
C. ‘Arabia maxima’	18	18	18	18	18	18	18	18	18	18	19	0.498	18.200
A3b. ‘Kulbicki et al.’	18	18	18	18	18	18	18	18	18	18	20	0.498	18.200
L. ‘Arabia dividua 2a’	19	18	18	18	18	19	19	19	19	19	20	0.485	1.000
F. ‘Arabia minima 1c’	19	18a	18a	18a	18a	18a	18a	18a	18b	19	19	0.484	2.300
A2b. ‘Briggs & Bowen’	19	18	18	18	18	18	19	19	19	19	19	0.427	0.900
I. ‘Arabia dividua 1b’	19	18	18	18	19	19	19	19	19	20	20	0.401	3.500
J. ‘Arabia dividua 1c’	20	18	18	18	19	19	19	19	19	20	20	0.394	2.300
K. ‘Arabia dividua 1d’	20a	18	18	18	19	19	19	19	19	20a	20b	0.387	3.700
H. ‘Arabia dividua 1a’	19	18	18	18	19	19	19	19	19	19	20	0.320	6.700
A2a. ‘Briggs & Bowen’	19	18	18	18	18	19	19	19	19	19	19	0.302	5.500
B. ‘Arabia classic’	18	18	18	18	18	18	18	18	18	19	20	0.301	12.700
E. ‘Arabia minima 1b’	19	18	18	18	18	18	18	18	18	19	19	0.247	10.900
A3a. ‘Kulbicki et al.’	20	18	18	18	18	18	18	18	18	20	20	0.247	10.900
D. ‘Arabia minima 1a’	19	18	18	18	18	18	18	18	18	19	20	0.212	14.100
A3c-d. ‘Kulbicki et al. ‘ ^3^	18	18	18	18	18	18	18	18	18	18	18	-	-

^1^ Socotra Archipelago (Soc), Gulf of Aqaba (GoAq), Northern and Central Red Sea (NC RS), Southern Red Sea (S RS), Western Gulf of Aden-Djibouti (W GoA), Eastern Gulf of Aden extended (E GoA ext.), Central Oman (CO), Gulf of Oman (GoO), Arabian/Persian Gulf (AG), and “Somali Current Coast” (SCC) (compare Material and Methods).

^2^ The suffix ‘p’ that indicates the modified province-level enumeration in the text is omitted in the Table 1.

^3^ Combinations A3c-d (based on Kulbicki et al. 2013) included for completeness; no ANOSIM values available.

In order to frame the analyses of the Arabian ecoregions at larger spatial scale for discussion purposes, joint resemblance patterns were inferred for the countries and main island groups of the western, central and northern Indian Ocean. The additional species lists for the 11 additional geographic units were primarily sourced from Fishbase [45]; critically reviewed and consolidated according to the literature and the authors’ expert knowledge. The resulting overall checklists comprise 604 species for the data set of eight key families and 1292 species for the complementary data set of twenty families (S1 Data).

## Results

### Subregional resemblance patterns

The statistical validity of the designated ‘basin groups’ Northern Indian Ocean (NIO, ~Socotra Archipelago), Gulf of Aden (GoA, ~Belhaf Headland), and Southern Red Sea (SRS, ~Kamaran Archipelago and Hanish Archipelago) obtained from ANOSIM tests was significant (Global R: 0.967, *p* = 0.00001).

**Fig 3:**
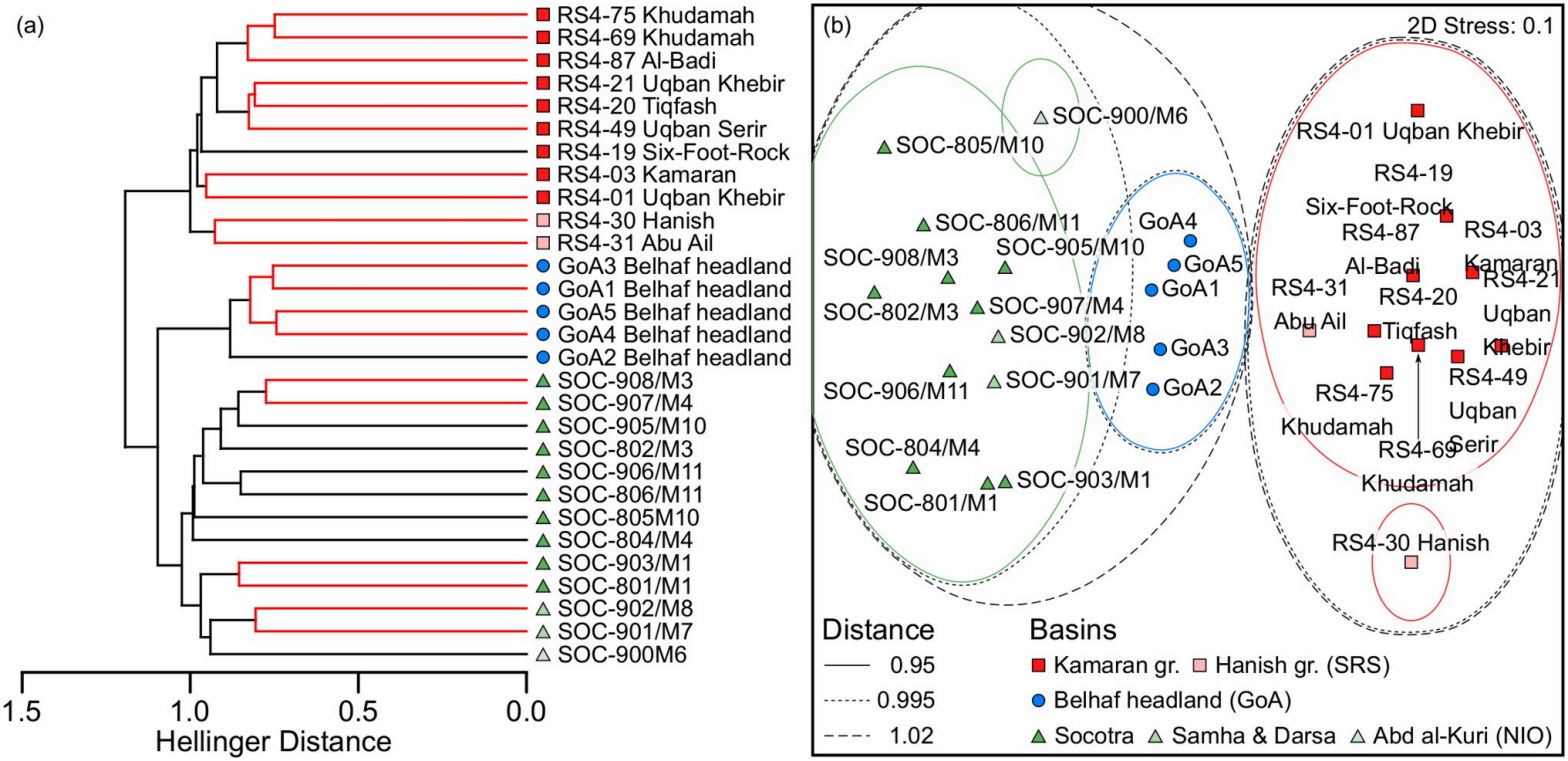
Incidence-based resemblance pattern of 29 subregional semi-standardized fish survey sites (belt transects and detailed fish inventories of near-similar survey effort, data of transects presence-absence transformed), composed of 13 fish transect sites on Socotra Archipelago of 2003 and 2007, 5 fish transect sites on Ras Belhaf, eastern Yemen, of 2005, and 11 survey sites including 7 inventories and 4 transect sites in the Yemen Red Sea from 2003 and 2004, with symbols representing *a priori* basin group designation overlaid; comparing (a) a dendrogram plot of a hierarchical agglomerative cluster analysis of 403 recorded species according to Hellinger distance (within-cluster data structure without SIMPROF support indicated in red), and (b) a non-metric multidimensional scaling analysis, based on data of the 8 key families only from the same data set. See Fig 2 for locations.

The corresponding resemblance of these 29 samples based on Hellinger distance is shown in the dendrogram plot of the hierarchical agglomerative cluster analysis (Fig 3a). It is congruent with the *a priori* basin group designations in that the samples from each ‘basin’ form distinct and statistically significant resemblance clusters, whereby data structure within them is partly not statistically supported towards the terminal branches.

Similar resulting patterns were obtained by reducing the input data set to the species of the eight key families, yielding a significant ANOSIM (Global R: 0.942, *p* = 0.00001). The corresponding resemblance is shown in the non-metric multidimensional scaling plot (Fig 3b). It has a relatively low stress (0.1) and reflects that NIO and GoA combine in the corresponding dendrogram before jointly combining with SRS (compare also S1 Fig).

The distance, SIMPROF and nMDS Stress values are provided in S3 Data.

### Regional resemblance patterns and alternative ecoregional arrangements

The statistical validation of 28 plausible *a priori*-defined ‘province-level combinations’ of the 10 Arabian ecoregional units (and Kenya) obtained from ANOSIM tests are listed in Table 1.

The province-level Combination A1 (‘Spalding et al.’, MEOW) only ranked 10^th^ in terms of Global R (Table 1). The nine more valid (in terms of Global R while being significant) province-level combinations separate the Socotra Archipelago from province-level combinations involving the Red Sea ecoregions, thus invalidating P18 of Spalding et al. (2007) [38] in terms of fish faunal resemblance. All of those nine combinations separate the Archipelago also from province-level combinations involving the Arabian/Persian Gulf, and eight of them from the Gulf of Oman. The province-level Combination A2b (‘Briggs & Bowen’, variant 2), while being significant, ranked 19^th^ only in terms of Global R. The Combination A2a (‘Briggs & Bowen’, variant 1) ranked 24^th^ in terms of Global R and is insignificant (Table 1). The province ‘Western Indian Ocean’ *sensu* Briggs and Bowen (2012) [39] therefore appears to be invalid. The province-level Combinations A3b and A3a (‘Kulbicki et al.’, b and a) do not validly reflect the distributional ichthyogeography of “Arabia” either, ranking 15^th^ and 26-27^th^ (along with Combination E.), respectively, in terms of Global R while being insignificant (Table 1).

Of the 10 most valid province-level combinations six place the Socotra Archipelago in a common province-level unit with the Somali Current Coast, and three place it into a common province-level unit with the Eastern Gulf of Aden extended and Central Oman. The nine most valid combinations thus disaggregate E89 (Gulf of Aden), and by consequence disaggregate also P18 (Red Sea and Gulf of Aden) in terms of fish faunal resemblance. In seven out of those 10 most valid combinations, the Arabian/Persian Gulf is placed into a common province-level unit with the Gulf of Oman, and in three it represents a province-level unit of its own.

The most valid province-level Combination U (‘Arabia dividua 4b’) has both the highest Global R (0.975) and the highest significance level (*p* = 0.00003) (Table 1). It disaggregates E89 (Gulf of Aden), and P18 (Red Sea and Gulf of Aden) by combining the Socotra Archipelago with the Eastern Gulf of Aden extended and Central Oman and by assigning the Western Gulf of Aden together with the Red Sea ecoregions into one putative province-level unit. Thereby, also P19 (Somalia/Arabian) is broken up, and further disaggregated by assigning the Arabian/Persian Gulf and the Gulf of Oman to a separate province-level unit, and the Somali Current Coast and Kenya to a separate unit. Therefore, both MEOW provinces P18 and P19 appear to be invalid in terms of fish faunal resemblance. The second most valid province-level Combination Z (‘Arabia dividua 5’) has a Global R of 0.957 (with *p* = 0.00009). It assigns the Socotra Archipelago to a province-level unit of its own, next to four additional province-level units (Table 1). The complete ANOSIM data including the pairwise test results may be found in S2 Data.

**Fig 4:**
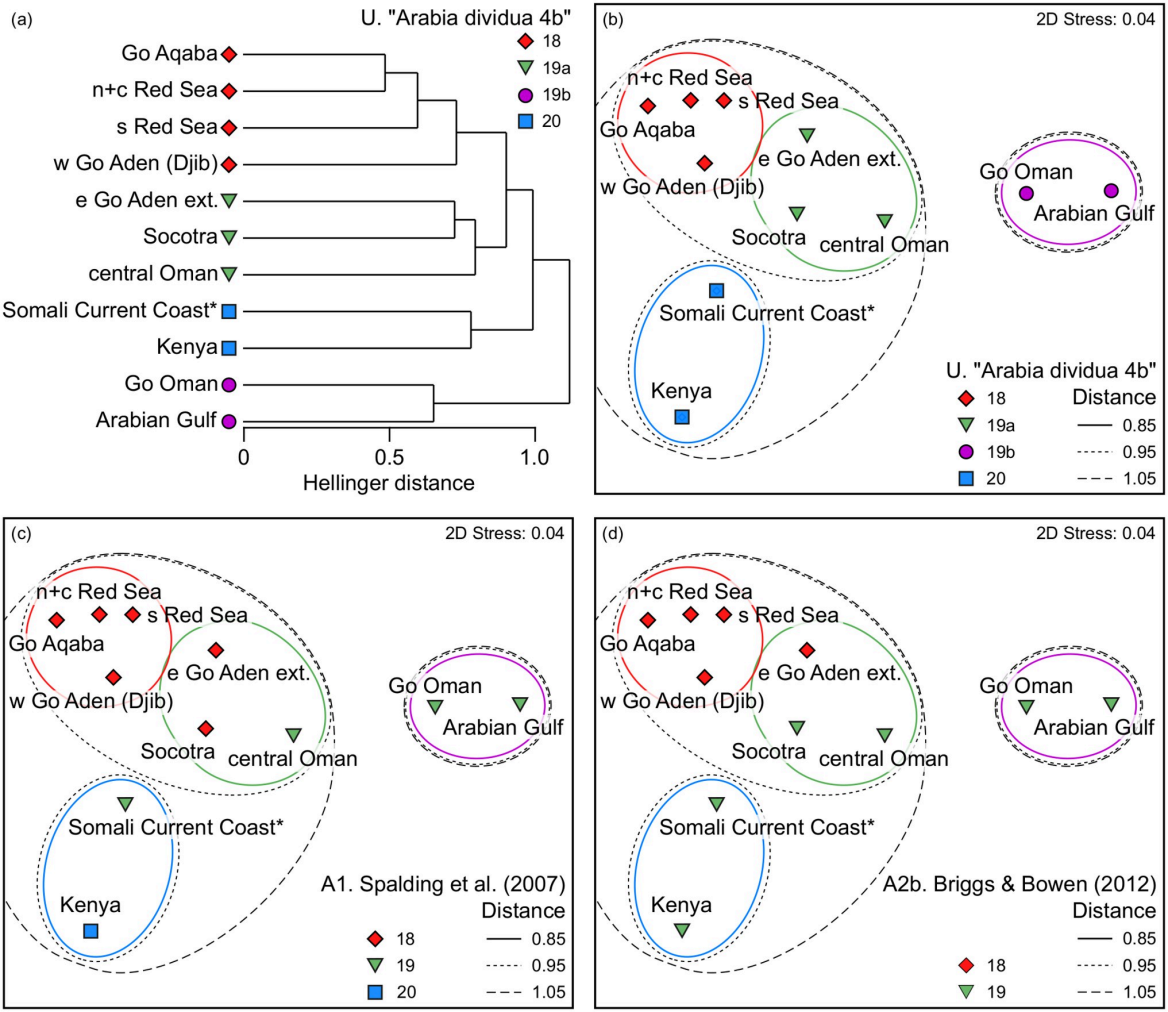
Resemblance pattern of 10 putative Arabian ecoregions and Kenya (8 key families, 404 species) based on Hellinger’s distance, represented as plots of the (a) dendrogram of the hierarchical agglomerative cluster analysis, and the (b) non-metric multidimensional scaling analysis (nMDS), both superposed with symbols representing the statistically (ANOSIM) most valid *a priori*-province-level Combination U (Table 1); compared with the same nMDS plot superposed with symbols representing the unsupported (ANOSIM) combinations (c) A1 following Spalding et al. (2007) and (d) A2b following Briggs and Bowen (2012) (province-level combinations A2a, A3b and A3a not shown because they are considered invalid according to ANOSIM; see S4 Fig).

The corresponding resemblance is shown in the cluster analysis plot (Fig 4a). It strongly reflects the biogeographical signal of the presumed ecoregions and primarily delineates two distinct and statistically significant clusters. One main cluster is formed by the Socotra Archipelago (e89c), the Eastern Gulf of Aden extended (e89b) and the Central Oman (e92), whereby the two former ecoregions are more closely related to each other than to the latter. The second main cluster is formed by the Gulf of Aqaba (e87a), the Northern and Central Red Sea (e87b), the Southern Red Sea (e88) and the Western Gulf of Aden (e89a), whereby the Gulf of Aqaba is closely related to the Northern and Central Red Sea. Jointly the “Socotra cluster” and the “Red Sea cluster” form a 2^nd^ order cluster which in combination with the Somali Current Coast (e93) and Kenya (which are part of a large cluster covering most of the remaining Western and Northern Indian Ocean; compare with Fig 8) form a 3^rd^ order cluster excluding as outgroup cluster the Arabian/Persian Gulf (e90) and Gulf of Oman (e91). The corresponding nMDS plot (Fig 4b) projects the multi-dimensional affinities onto a two-dimensional plain at a low stress (0.04). Separate resemblance analyses were also conducted for the eight key families individually and the ensuing dendrograms plots are provided in S2 Fig. The distance, SIMPROF and nMDS stress values are provided in S3 Data.

Superposing symbols corresponding to the province-level designations of the ecoregions according to the statistically most valid ANOSIM Combination U on the cluster dendrogram and nMDS plot shows the close concordance of the results (Fig 4a and 4b). Conversely, superposing province-level designations on the same nMDS plots according to the combinations A1 (‘Spalding et al.’, Fig 4c) and A2b (‘Briggs & Bowen’, Fig 4d) illustrates their low validities, with the colour coding not matching the ordination.

Analysing with SIMPER the province-level grouping of the putative ecoregions of the statistically most valid Combination U (Table 1) revealed the following within-group similarity values: group 18 (78.49), group 19b (76.92), group 19a (69.36), and group 20 (68.53). The highest dissimilarity was found between p19b and 20 (74.46) and p18 and p19b (69.29), followed by p19a and p19b (57.32), p18 and p20 (52.39), p19a and p20 (47.88) and p19a and p18 (41.49).

**Fig 5:**
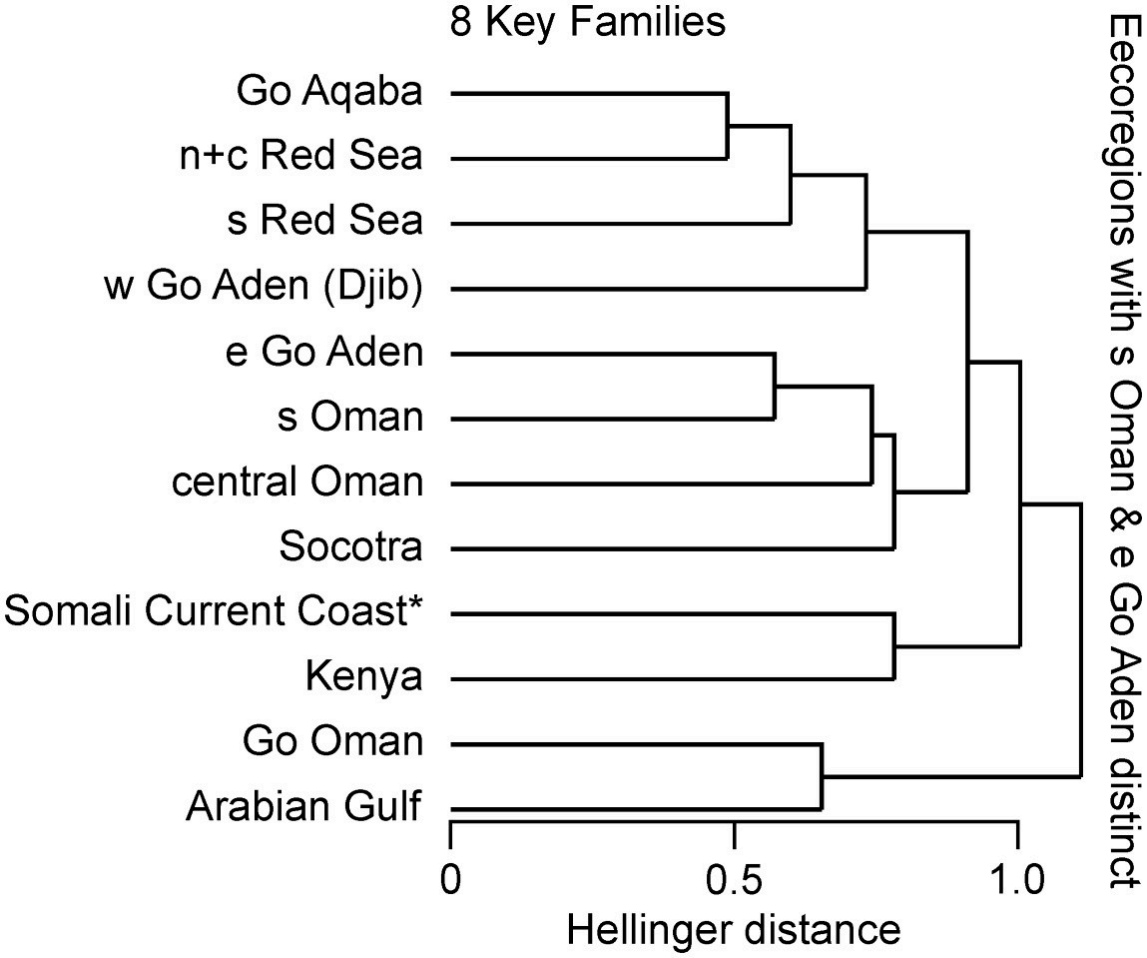
Resemblance pattern of 10 putative Arabian ecoregions and Kenya (8 key families, 404 species) based on Hellinger distance, represented as plot of the dendrogram of the hierarchical agglomerative cluster analysis, according to separate checklists for the eastern Gulf of Aden (e Go Aden) and southern Oman (s Oman), verifying the proposed Eastern Gulf of Aden extended (e Go Aden ext.) ecoregion used in the analysis presented in Table 1 and Fig 4.

The results of the complementary resemblance analysis of eight key families of according to separate checklists for the eastern Gulf of Aden and southern Oman conform closely to the main analysis. The “Socotra” cluster within wider Arabia is maintained and statistically significant. The Eastern Gulf of Aden is most proximate to Southern Oman, then linking with Central Oman in forming a mainland cluster, before uniting with Socotra (Fig 5). The distance and SIMPROF values are provided in S3 Data.

**Fig 6:**
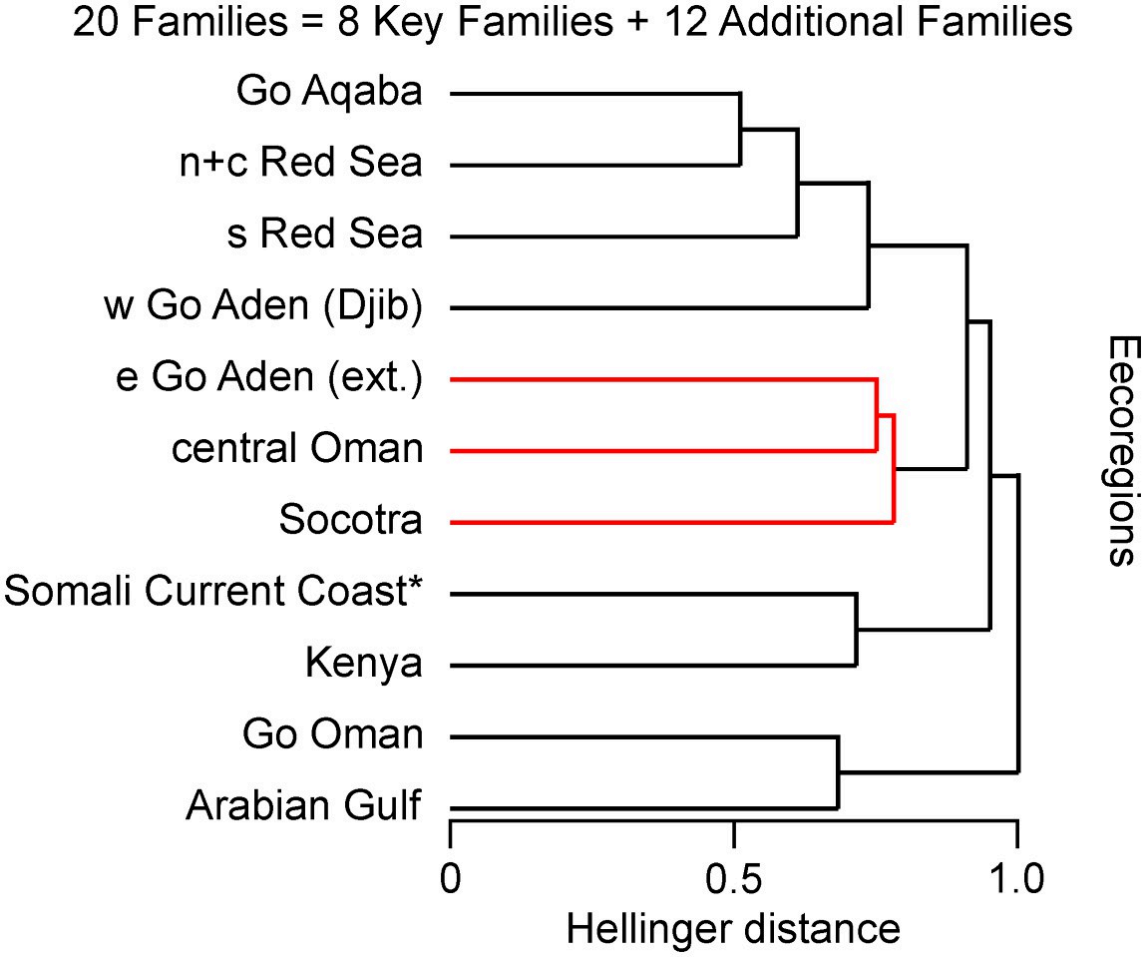
Resemblance pattern of 10 putative Arabian ecoregions and Kenya (20 core families, 898 species) based on Hellinger distance, represented as plot of the dendrogram of the hierarchical agglomerative cluster analysis, verifying the analysis of 8 key families shown in Fig 4 (within-cluster data structure without SIMPROF support indicated in red).

The results of the complementary resemblance analysis of twenty core families, adding 494 species (increase by 122%) conform closely to the main analysis. The “Socotra” cluster within wider Arabia is maintained. The within-cluster relations of Socotra to the Eastern Gulf of Aden extended are more distant with the latter being closer to Central Oman, whereby this structure is not statistically significant (Fig 6). The distance and SIMPROF values are provided in S3 Data.

## Discussion

**Fig 7:**
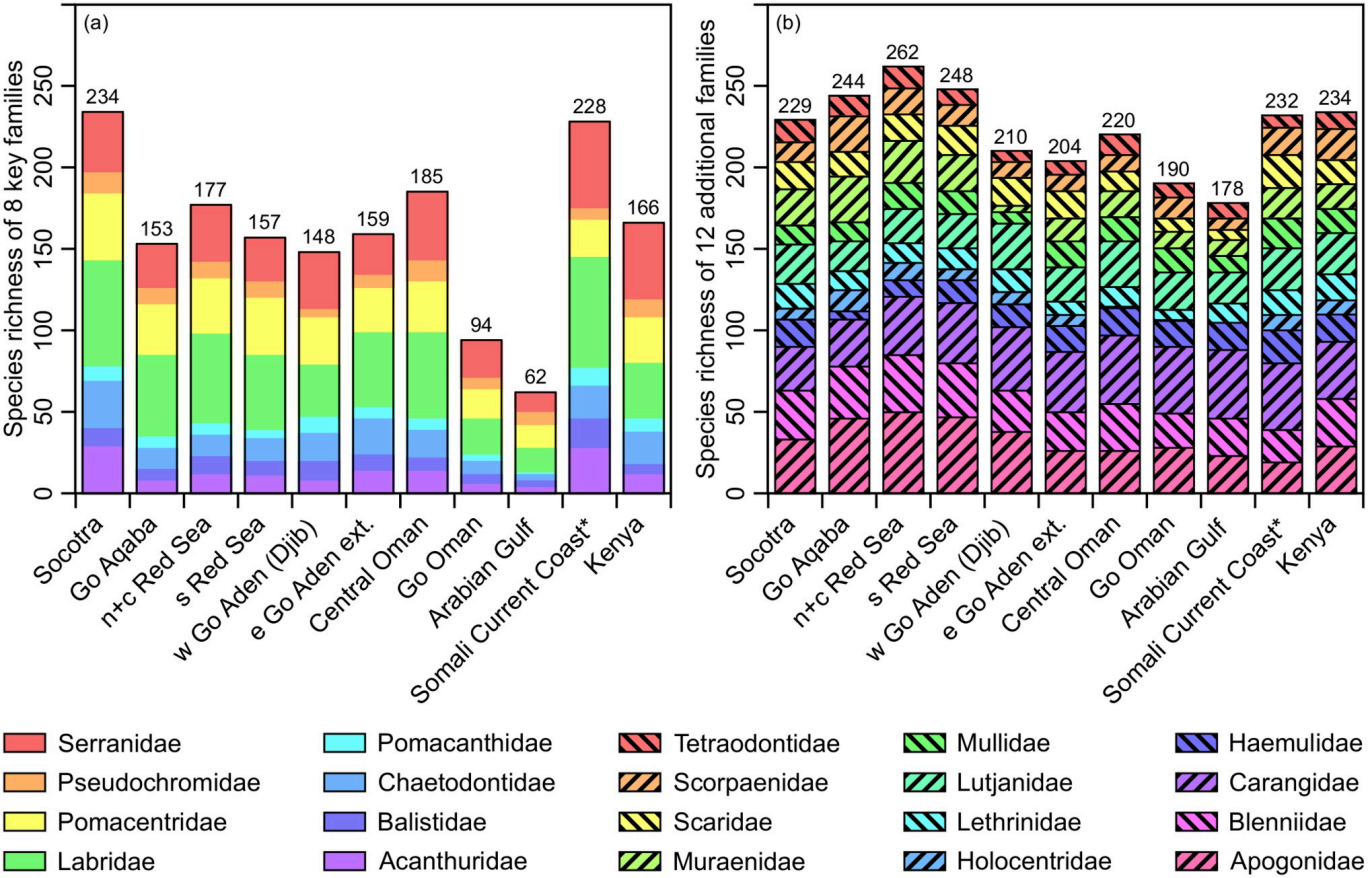
Comparison of species richness in 10 putative Arabian ecoregions (modified from Spalding et al. 2007), and Kenya as external reference (* surrogate for the eco-regions ’Central Somali Coast’ and northern part of ’Northern Monsoon Coast’ of Spalding et al. 2007), according to (a) 8 key families (modified from Zajonz et al. 2019), and (b) 12 additional core families (this study).

A recent study by Zajonz et al. (2019) [4] showed that the coastal fish diversity of the Socotra Archipelago is the highest among comparable Arabian biogeographic units (Fig 7a and 7b). Kemp (1998) [5] listed 49 species in four key families investigated primarily (Chaetodontidae, Pomacanthidae, Acanthuridae and Balistidae), including 13 chaetodontids. Based on Zajonz et al. (2019) [4], the present study recognizes 78 species in these families, including 29 chaetodontids, underscoring the need for an updated biogeographic analysis.

Frequencies of *a priori* defined global distribution ranges were presented by Zajonz et al. (2019) [4], covering 658 species from the Archipelago, supporting the perception of the island group as a “zoogeographic crossroads” and a biogeographic “stepping stone” (e.g., [5, 9, 11, 16, 18, 63]). Similar distribution frequencies are observed from the reduced data set of eight key families primarily assessed in the present study (S4 Data, S4 Fig).

### Subregional resemblance patterns

The coastal fish assemblages of Socotra show a modest intra-archipelagic (bio-) geographic resemblance structure according to island groups [4]. The present study demonstrates that it forms an archipelagic assemblage cluster that is distinct from neighbouring assemblages in the southern Arabian region.

Both the R values of the ANOSIMs and the distance values of the cluster analyses suggest that the assemblages of the Socotra Archipelago (‘NIO’) are most closely related to those of the Belhaf headland (‘GoA’). The latter are closer to the assemblages of the southern Red Sea (‘SRS’) than those of the Socotra Archipelago. The results based on the reduced species dataset closely match those obtained from the full dataset, confirming the eight families as suitable ichthyogeographic "proxies".

The marked ichthyogeographic affinity of Socotra Archipelago to a putative "South Arabian Region" postulated by Kemp (1998, 2000, S4 Fig) [5, 8] was based on relatively limited faunistic data and not supported by a joint resemblance analysis of both areas. The present subregional assemblage analysis provides explorative statistical evidence for the affinity between Socotra Archipelago and the mainland coast of southern Arabia. Kemp (2000) [8] also indicated a faunal break in the Hadramaut region west of Al-Mukallah, limiting the putative “South Arabian region” (with strong affinities to Socotra) in westward direction. The present study links the Socotra assemblages to sites at Belhaf headland, thus to the western side of the postulated faunal break. Kemp (2000) [8] explained the putative faunal break as related to an upwelling and productivity boundary at about the position of Burum (east of Belhaf). There is evidence, however, that the south Arabian upwelling areas reach until Belhaf headland, as discussed further below. The hypothetical faunal break west of Al-Mukallah appears of limited importance, and a main break is rather located further west. Combining the results of [5, 8, 37] and the present study, the presumed "South Arabian Region" seems to span an area from southern Oman to about the Belhaf headland.

With regard to the provincial boundaries proposed by Spalding et al. (2007, MEOW; Combination A1) [38], the present subregional analysis suggests that the Socotra Archipelago should not be included in their province P18. It clusters to a greater extent with sites which are not assigned to the Gulf of Aden ecoregion (E89) of this province, i.e. to the neighbouring E92 in province P19. Alternatively, the boundary delineation of the Gulf of Aden ecoregion needs to be adjusted (further reasoning and adjustments are proposed below).

With regard to the provincial boundaries proposed by Briggs and Bowen (2012; Combination A2) [39], the present subregional analysis suggests to adjust the delineation of the boundary between their Red Sea province and Western Indian Ocean province. The delineation at Ras Fartak should shift to a position west of Belhaf (further reasoning and adjustments are proposed below).

With regard to the proposed provincial boundaries associated with Combination A3a of Kulbicki et al. (2013; Fig 4(a), no boundary lines drawn) [40], the present subregional analysis suggests that the separation of Socotra Archipelago from the Arabian Peninsula at the provincial level is questionable. No inferences can be made regarding the boundaries associated with the combinations A3b and A3c-d.

### Regional resemblance patterns

The dendrogram of the main analysis (Fig 4a) illustrates four basic regional resemblance clusters at a distance of 0.85. The low distance between the Gulf of Aqaba (e87a) and the Northern and Central Red Sea (e87b) suggest that both form a common ecoregion. According to the most valid ANOSIM Combination U, the low Hellinger distances and high SIMPER similarity values for the putative province-level units p18 (Red Sea and Western Gulf of Aden) and p19b (Arabian/Persian Gulf and Gulf of Oman) are compelling. By comparison, the SIMPER values for p19a (Socotra, Eastern Gulf of Aden ext. and Central Oman) and p20 (Somali Current Coast and Kenya) are lower. This is likely in part due to the transitional position of Socotra Archipelago at “crossroads” [11, 29] having substantial affinities in either direction, north and south. The weaker similarities also correspond to, first, the 2^nd^ most valid ANOSIM Combination Z that designates the Archipelago as a province of its own, second, the relatively high statistical validity granted to ANOSIM combinations that assign the Archipelago to a common province-level unit with the Somali Current Coast, and, third, the low SIMPROF support to the within data structure of the “Socotra cluster” of the complementary analysis of 20 families (Fig 6). The respective nMDS plot (Fig 4b) illustrates this position in a highly intuitive way, placing the Archipelago at the centre of a triangle formed by the ecoregions included in this study in two-dimensional space. The plot puts it in almost equidistant position to the nearest ecoregions Eastern Gulf of Aden ext., Central Oman and Somali Current Coast. The comparatively weaker similarities are likely also computationally influenced by joint species absences among the p19a and p20, owed to a distributional gap of several wide-ranging species in the upwelling areas of southern Arabia [5, 13, 30, 64]. The separate resemblance analyses conducted for the eight key families individually (S2 Fig) provide additional insights into the underlying distributional patterns.

The dendrogram of the complementary analysis according to separate checklists for the Eastern Gulf of Aden and Southern Oman (Fig 5) supports them being treated as one putative ecoregion in the main analysis. The dendrogram of the complementary analysis according to twenty families (Fig 6) largely corroborates the main analysis. The input species number is increased by 112%, covering more than half of the coastal fish species of Arabia (~1700 species estimated by the authors), without major changes to the clustering pattern. This also grants confidence into the representativeness of the eight key families selected for the main analysis. The variation of the relatedness patterns within the “Socotra cluster” between the three analyses suggests the need for additional, spatially augmented faunistic data from those areas.

**Fig 8:**
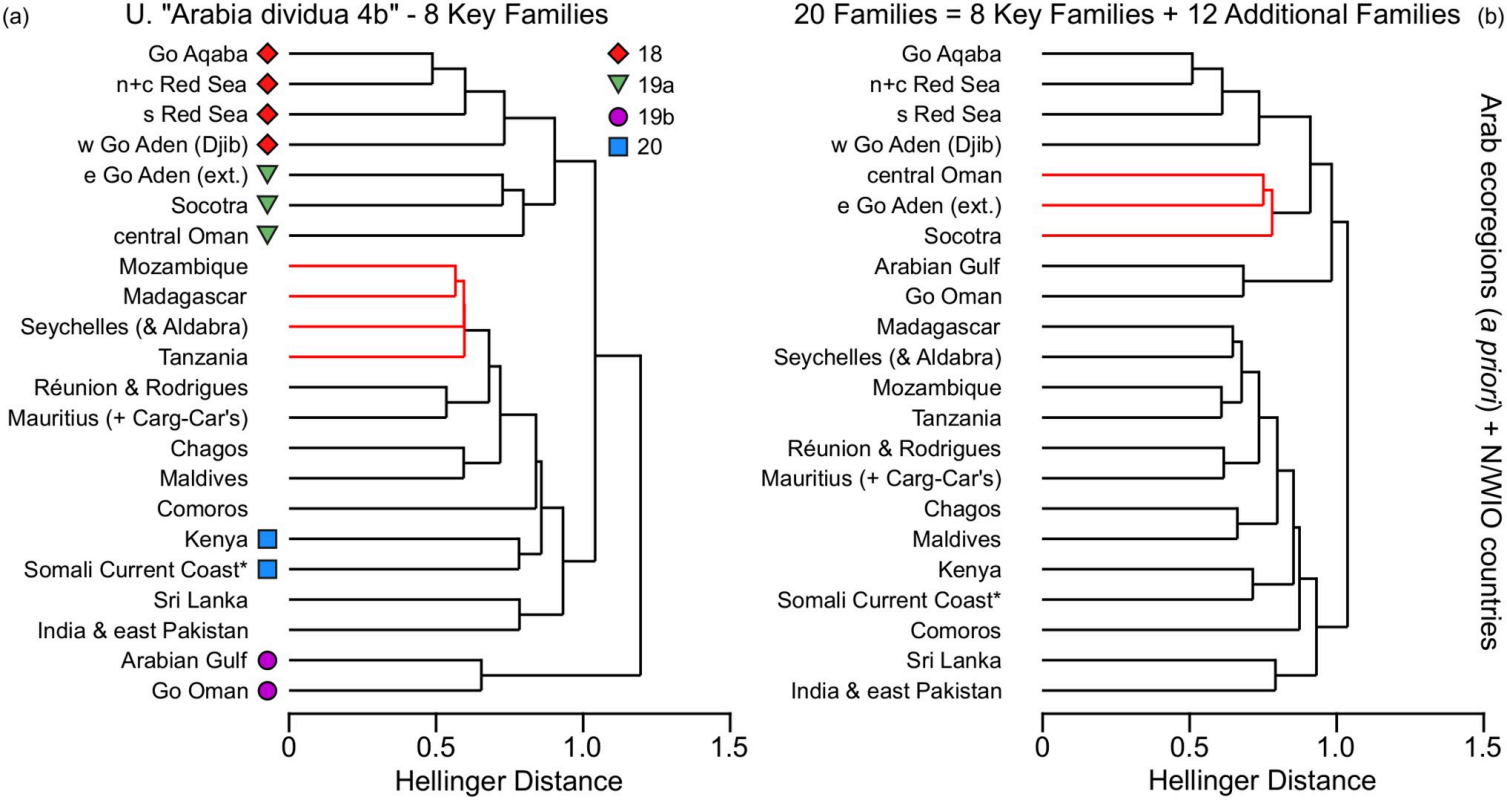
Resemblance pattern of 10 putative Arabian ecoregions, and countries and island groups of the wider Northern, Western and Central Indian Ocean. The dendrogram (a) of the hierarchical agglomerative cluster analysis of 604 species in 8 key families is compared to the dendrogram (b) of the hierarchical agglomerative cluster analysis of 1292 species in 20 core families; with (a) superposed with symbols representing the statistically (ANOSIM) most significant *a priori* Combination U of province-level designations for Arabian ecoregions (compare Fig 4a) (within-cluster data structure without SIMPROF support indicated in red).

Framing the analyses of the Arabian ecoregions the resemblance patterns inferred from the western, central and northern Indian Ocean (604 species, Fig 8a) corroborate the main faunal clusters resulting from the Arabian analysis alone (Fig 4a and 4b). The dendrogram plot indicates three main clusters at a Hellinger distance of about 1; (a) Red Sea, Gulf of Aden, Socotra and western Arabian Sea, (b) Arabian/Persian Gulf and Gulf of Oman, (c) remaining western, central and northern Indian Ocean, including the ‘Somali Current Coast’ and ‘Kenya’, with India and Sri Lanka forming a distinct subcluster. These resemblance patterns are broadly confirmed by the complementary analyses of the distribution of 20 families (1292 species, Fig 8b) and e.g. of chaetodontids (58 species, S4 Fig). The resemblance patterns of the individual families vary considerably and for example the one of the Labridae place Socotra in the (c) cluster (S4 Fig). As in the Arabian analysis alone, the cluster (b) is identified in the analysis of eight key families as “outgroup” to all other geographic units, being more distant to other Arabian units than the Somali Current Coast and Kenya. The cluster (b) is realigned with “Arabia” in the expanded analysis because the latter two sites relate to Western Indian Ocean sites they are even closer to than to Arabian sites. This outcome is also congruent with the high SIMPER dissimilarity values to all other putative province-level units in the main analysis. It tentatively suggests a high difference of the Arabian/Persian Gulf and Gulf of Oman to other parts of Arabia and the Western Indian Ocean and should be validated in future analyses. Such a distinction is also supported by the ecoregional analysis of zooxanthellate Scleractinia of Veron et al. (2015) [65]. In a clustering dendrogram of the western and central Indian Ocean presented by those authors, the Arabian/Persian Gulf had most distant affinities to any other circum-Arabian ecoregion. It is beyond the scope of this paper to discuss the fish resemblance patterns at this geographic scale in further detail.

In comparison with Kemp [5, 8] the affinities of the Socotra Archipelago to a “South Arabian Region” are largely confirmed also by the present regional resemblance analyses. The relatively close relationship to “Oman” [5] (S4 Fig) is confirmed for Southern Oman and Central Oman, but not for the Gulf of Oman. Also the study of Burt et al. 2011 [66] clearly separates fish communities at the Dhofar coast from those in the Gulf of Oman and the southern Arabian/Persian Gulf. A relative proximity to the Arabian/Persian Gulf ([5], Fig 5) is rejected. A putative zoogeographic barrier south of Socotra ([5], Fig 2) and the distance (dissimilarity) to East Africa ([5], Fig 5) are most probably much less pronounced than suggested by Kemp, considering the strong affinities of the Socotra Archipelago to Somalia and East Africa, both in terms species distribution frequencies ([4]; S4 Fig) and statistical evidence (Table 1); as shown here above. The affinity of the southern Red Sea ecoregion to the western Gulf of Aden is strongly supported (see also [13, 30]). The role of the sill of Bab al-Mandab (*sensu* Springer and Williams 1994, in Bellwood and Wainwright 2002) [67] as a barrier to coastal fish dispersal appears to be limited.

The interpretation of the results needs to be considered against the backdrop of the following issues: (1) This study primarily analyses distributional data of eight key families. Limitations to field work and data coverage framing ecoregional analyses in the region of concern are substantial. While compiling quality checklists for the Socotra Archipelago, the northern and central Red Sea and the Arabian/Persian Gulf was feasible, it was much less so for southern Arabia and north-eastern Africa of which large parts have been barely accessible during the past two decades. No comprehensive ichthyofaunal accounts exist for them and only few credible species records have been published [8, 16, 36, 68]. The resulting checklists bear strongly on data of the authors which are published here for the first time. Global biodiversity data repositories like GBIF, OBIS or FishBase often hold spatially incomplete data of very mixed taxonomic quality as pointed out by Robertson (2008) and Chollett and Robertson (2020). Those authors found “large-scale errors in over a third of the species and genera, in nearly two-thirds of the families” [69] pertinent to the Caribean, and that “spatial [data] biases produce artefactual variation in patterns of species turnover and delineation of bioregions” [70]. A similar if not more severe caveat exists for southern Arabia. Focussing on eight key families especially seeks addressing the eminent requirement to feed well curated data into the biogeographic analyses. The critical review of tropical reef fish evolution by Cowman (2014) [71] focussed on nine characteristic reef fish families. A predictor of theoretical reef fish diversity in a given area was developed by Allen and Werner (2002) [72] based on the richness of six conspicuous reef fish families. While some resemblance indices are robust to a certain level of artefactual absences [53, 73] the sampling bias and overall taxonomic uncertainty across the Arabian ecoregions is substantial. The probability of joint presences, thus of computational key information, is seriously compromised unless checklists are meticulously prepared. In their biogeographic study of reef fishes in the South Atlantic Joyeux et al. (2001) [74] applied a selection of eight families for those reasons, focussing on 35 species. Investigating the same study subject Floeter and Gasparini (2000) [75] analysed 40 families and likely 300-400 species (checklist undisclosed) while Pinheiro et al. (2018) [76] analysed 405 resident reef species out of a checklist of 733 coastal species. The data of eight key families analysed in the present study is well comparable with those studies. The complementary analyses comprise twenty of the twenty-two most species-rich families from the Archipelago [4] (excluding Gobiidae for bearing a high taxonomic error and Carcharhinidae for being biogeographically uninformative at the spatial scale of the study [77]). The additional families more than double the input species numbers and comprise in excess of 50% of the total species richness expected from each of these areas. (2) The entire coast of Somalia is not well researched; the data are partly interpolated and spatially not well resolved (as in all studies compared with). No definite inferences can yet be drawn about the ecoregional delineation of the south-eastern Gulf of Aden and the eastern coast of Somalia. A surrogate dataset was constructed for the Central Somali ecoregion (E93 of MEOW), which might not exactly reflect its northern and southern boundaries. (3) The north-western Gulf of Aden from about Aden to Belhaf Headland is data deficient; the presumed boundary between the north-western and the north-eastern Gulf of Aden ecoregions at Belhaf needs to be confirmed following more field research. (4) The presumed position of the eastern boundary of the extended eastern Gulf of Aden ecoregion at the Hallaniyat Islands also requires confirmation following more field research. (5) The Gulf of Oman data are preliminary because no separate authoritative species account for this basin exists to date. (6) Methodologically each of the three main studies tested here (Fig 1a–1c) grant a certain informational weight to “the level of species exclusive to an area considered (endemics)” (Kulbicki et al. 2013 [40]). The present study does not do so because the levels of endemism in parts of the study area are not yet well established. Besides endemicity Mouillot et al. 2013 [78] also highlighted the value of considering the turnover component of resemblance measures in order to capture nestedness. Both endemicity and nestedness shall be reflected in a forthcoming study involving additional organism groups (e.g. [79]). (7) While it is beyond the scope of this study to comprehensively review the phylogeographic literature, no available phylogeographic data (i.e. lately [32, 34, 35, 80, 81]) contradict the results, to the best of the authors’ knowledge.

Pertinent to the working hypotheses the results are construed as follows: (i) the wider Gulf of Aden does not represent a consistent ecoregion in terms of fish assemblage composition because it falls into two to three ecoregion-level and two province-level units; (ii) the eastern Gulf of Aden is more closely related to southern Oman than to its western part and southern Oman is at least as closely related to it than to central Oman, whereby additional faunistic studies are desirable in this region; and, (iii) the Socotra Archipelago should not share a province-level unit with Red Sea ecoregions; a redelineation of the ecoregions in southern Arabia is required, that the Archipelago may ultimately represent an ecoregion of its own is indicated by its comparatively large distances to other putative ecoregions within its cluster throughout the various regional analyses, and by the second-most valid ANOSIM which assigns it a province-level of its own.

**Fig 9:**
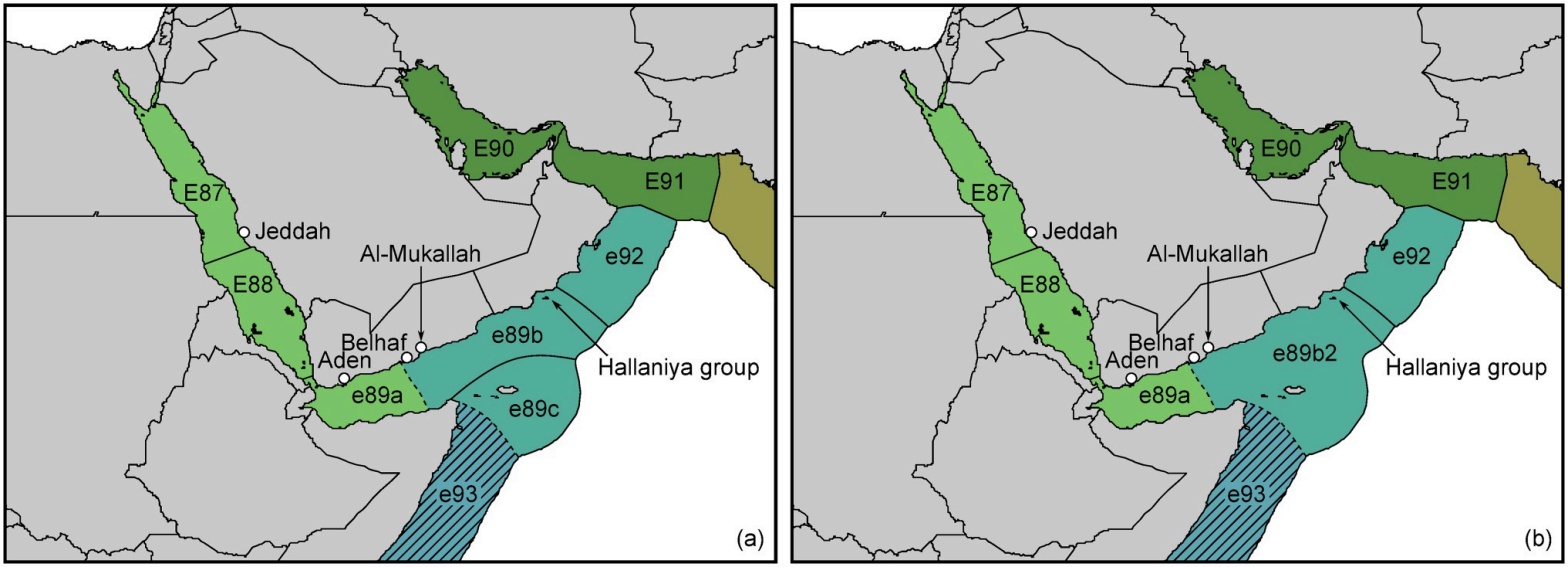
Provisional alternative delineations of Arabian ecoregions and provinces, modifying Spalding et al. (2007) by (a) tripartitioning ecoregion E89, designating the Socotra Archipelago a distinct ecoregion (e89c) besides the Western (e89a) and extended Eastern Gulf of Aden (e89b), and modifying ecoregion E92 (and a slight modification of ecoregion E93); (b) bipartitioning ecoregion E89 into a Western Gulf of Aden (e89a) and a common ecoregion for the extended Eastern Gulf of Aden and Socotra (e89b2), and modifying ecoregion E92 (amended provinces are indicated by shared colours, and boundary delineations that require further confirmation are indicated by dashed lines). (a, b: Baseline map sourced 2019 from Natural Earth, free vector and raster map data <naturalearthdata.com> with no copyright restrictions. Ecoregion boundaries redrawn and partly modified from GIS data accompanying Spalding et al. (2007), sourced 2012 from <conserveonline.org>, with free permission for scientific use and reproduction (to date only available from <worldwildlife.org>), colours modified).

An update to the realignment of marine biogeographic regions and provinces of Briggs and Bowen [39] is suggested (see also [82]), in considering that these authors use two hierarchical classification levels only (Fig 1b). It envisages placing the Arabian Sea coast of Oman (composed of southern and central Oman) and Socotra Archipelago in the same province as the Red Sea and Gulf of Aden (composed of the western and eastern Gulf of Aden). The northerly boundary of the present ‘Western Indian Ocean’ province should locate somewhere at the east coast of Somalia (precise location to be determined). An update to the global hierarchical delineation of reef fish regions of Kulbicki et al. (2013) [40], notably the arrangement favoured by them among their alternative study results (Combination A3a, Fig 1c and 1d) should consider assigning the Socotra Archipelago to their ‘North-western Indian Province’. This move would also be supported by Borsa et al. (2015) [83]. These authors, in using basically the same data set as Kulbicki et al. (2013) [40], assigned the Socotra Archipelago to a pan-Arabian ‘North-western Indian’ province and thus separated it from the Somali Current Coast and the southward coasts of East African. In addition, either concept should consider the designation of the Arabian/Persian Gulf, possibly in combination with the Gulf of Oman, as entity of its own, unless future studies satisfactorily confirm them constituting a circum-Arabian province. The MEOW scheme (Fig 1a) [38] requires substantial adjustments (compare [79]) if it were to satisfactorily capture the coastal ichthyography of “Arabia” resulting from the present analyses. Its ‘Gulf of Aden’ (E89) needed to be divided into two or three separate ecoregions. Two provisional alternative delineations are proposed in Fig 9 and discussed below. Each of those three eminent studies used its own method, and is not challenged *per se*. Spalding et al. (2007) [38] noted, with Wallace (1876) [84], that “*nothing like a perfect zoological division of the earth is possible …* “and implied welcoming revisions of the lines drawn by them.

### Alternative ecoregional delineations as onward working hypotheses

The present study revised the distributional ichthyogeography of the Socotra Archipelago and southern Arabia. It provides critical evidence to propose alternative delineations of the MEOW, if as ecoregional working hypotheses for future study. According to Lomolino et al. (2010) [85] ecoregions are biogeographic divisions of the Earth which seek to account for present distributions resulting from organisms interacting with their physical and biotic environment. The ultimate delineation and justification of the proposed ecoregions therefore require to relate the ichthyogeographic pattern to the distributions of other marine organism groups, and to the ambient physical, oceanographic and climatic conditions. Additional inferences might be drawn from the analyses of connectivity processes, dispersal capacities, and levels of endemicity and hybridization. In theory, an onward analytical bioregionalization study could start *de novo* without referring to the spatial templates of established concepts and pre-structured data sets (e.g. [86]). A temporally and spatially structured study design would ideally involve the repeated sampling of several key marine and coastal organism groups along with crucial environmental data. A relatively fine survey grid covered the shores of southern and eastern Arabia and the adjacent African coastline. Environmental sampling, both in situ and remote sensing, included geophysical parameters, e.g. width of the coastal shelf, sedimentation, main substrate, and oceanographic parameters, e.g., sea surface temperature and height, turbidity, and productivity based on chlorophyll-a and carbon-based productivity models. Especially the prevalence of seasonal upwellings is expected to structure coastal communities at the regional scale. Both abundance and incidence-based community patterns would be statistically identified and related to the environmental data, characterising spatially explicit combinations of relatively homogeneous physical and biological attributes. Zones of accelerated change inbetween them qualified as boundaries. Logistic and security constraints, however, are most likely to impede such a study design for southern Arabia for some time to come. A more conservative study approach would thus rather seek to iteratively verify individual hypothetical boundaries, within established biogeographic concepts. It resorts to quality checklists for biotic data and environmental variables sourced from remote sensors and databases. It identifies critical environmental variables which best explain competing putative distributional patterns derived from alternative pre-structured multi-taxa data sets.

Yet, there is already a substantial body of literature to embark from. It covers inter alia the ecological biogeography of other key taxa and the regional monsoon-driven oceanography and climate. The study of Zajonz et al. 2016 [15] already provided a first stab at referring fish assemblage structure to the dramatic seasonal fluctuations of key oceanographic parameters, notably temperature and biological productivity, as inferred from remote sensing data. Key considerations that accordingly helped shaping the novel ecoregional concept and support onward research are as follows.

The alternative delineations (Fig 9a and 9b) consider a ‘Western Gulf of Aden’ ecoregion (e89a) that extends in the north to the eastern end of Al-Ayn Bay at Belhaf headland; its south-easterly boundaries remain to be confirmed (compare Costello et al. (2017) [87] who draw the boundary between their realms no. 13 and 14 in the central Gulf of Aden). The ‘Eastern Gulf of Aden extended’ and the ‘Socotra Archipelago’ represent either two separate ecoregions (e89b and e89c) (Fig 9a) or a common one (e89b2) (Fig 9b), pending additional evidence, whereby in either arrangement, e89b or e89b2 likely extends eastward until the Hallaniyat Islands in southern Oman, following Kemp (2000) [8] and Kemp and Benzoni (2000) [37]. The south-western boundary of the ‘Western Arabian Sea’ ecoregion (e92, here ‘Central Oman’) should be arranged accordingly north of the Hallaniyat Islands. The selection of this boundary also appears to be justified by greater similarities of the oceanographic conditions found between Sharbatat Headland and Ras al-Hadd, as compared to the Dhofar coast. This is particularly obvious during the winter monsoon season (January-March) and the intermonsoon months (October-December) (see also [88–90]).

The proposed boundary between e89a and e89b (or e89b2, respectively) is also defined by the influence of the Indian south-west monsoon. The monsoon-driven coastal upwelling triggers primary productivities which sharply contrast between the western and eastern Gulf of Aden, as already noted by Kemp (2000, Plate 17) [8]. Remotely-sensed chlorophyll-a concentrations [15, 91] indicate that coastal waters are fertilized by nutrient-rich upwelling waters westward to Belhaf Headland. This corresponds to high inshore fish biomass concentrations around this coastal promontory (Zajonz unpublished data). Sea water parameters monitored *in-situ* at Belhaf Headland [92] and chlorophyll-a concentrations measured *in-situ* along the Hadramaut coast [91] strongly support these observations. The primary productivity is substantially lower from about the boundary line between e89a and e89b indicated in Fig 9 in westward direction during south-west monsoon season of June/July to October [12, 15]. This boundary line is congruent with the eastern margins of the Gulf of Aden ‘summer eddy’ [93], which blocks the westward propagation of eddies and currents originating from the Arabian Sea during the summer months [94]. Inversely, from November to February/March the waters of the inner (western) Gulf of Aden are more productive. Compared to the summer, the winter pattern is more dynamic and variably delineated by the margins between the Somali Current Ring, introgressing into the Gulf of Aden between Cap Guardafui and Abd al-Kuri [88], and the ‘anticyclone 1’ eddy [95]. Also physical connectivity simulations based on Lagrangian particle trajectories [96] appear to support such a boundary. Besides biological productivity these patterns obviously also reflect physical parameters like sea water temperature and wind speed, which likely influence the dispersal and distribution of marine organisms in the Gulf of Aden. Sea Surface Temperatures (SST) are substantially higher in the western Gulf of Aden during the south-west monsoon (e.g. [97]) than in the eastern Gulf of Aden, the 20 C thermocline is deeper in the eastern Gulf of Aden during the intermonsoon period March to June [93], and the wind stress is higher in the eastern Gulf of Aden during the north-west monsoon (e.g. [98]). How these highly dynamic environmental patterns interact spatio-temporally with the reproductive and dispersal traits of marine organisms, thus population genetics, is not well understood (see [99] for a first stab). The northern boundary between ecoregions e89a and e89b also in geomorphological terms seems to be reasonably located at the intersection of Belhaf Headland and Al-Ayn Bay, since it marks the beginning of more than 250 km of continuous coastline dominated by sand and other soft sediments in a westward direction.

The new ecoregions e89b and e89c, or e89b2, respectively, should be considered belonging to an ‘Eastern Arabian’ province (p19a) with the modified ecoregion e92 (‘Central Oman’), thus be separated from the present ‘Red Sea and Gulf of Aden’ province (P18). Whether the ‘Central Somali Coast’ ecoregion (E93) should be considered part of province p19a requires further investigation. The ‘Arabian/Persian Gulf’ ecoregion (E90) should not remain part of P19. It should form a province-level unit (p19b) either of its own (compare [65]) or, more likely, be merged with the ‘Gulf of Oman’ ecoregion (E91), pending more reliable species inventories for the latter basin. The proposal to assign E91 and E92 (the latter as e92) to two different province-level units is also supported by Schils and Wilson (2006) [100], who showed a pronounced macro-algae assemblage turn-over associated with the oceanographic conditions north and south of Ras al-Hadd. The surrounding MEOW provinces ‘Western Indian Ocean’ (P20), ‘West and South Indian Shelf’ (P21), and ‘Central Indian Ocean Islands’ (P22) are largely confirmed. The fact that they cluster altogether next to a cluster formed by the proposed Arabian province-level units p18 and p19a (Fig 9) could be captured by an additional hierarchical level ‘superprovince’ ranking in between ‘province’ and ‘realm’. While suggesting these amendments, it is well appreciated that the analysis underlying the MEOW scheme has been much wider in scope than the present study.

How populations on Socotra are connected through larval dispersal and recruitment with the surrounding mainland coasts is not well understood yet. A study from the Philippines [101] estimated “that 50% of [reef fish] larvae originating from a population would attempt to settle within 33 km, and 95% within 83 km”. Dispersal distances to connect from the Archipelago to populations in Somalia (~100 km from Abd al-Kuri) and especially Yemen (~330 km from Socotra Island) are larger. A related study [102] found close concordance of fish assemblage resemblance and dispersal models allowing to explain biogeographic patterns across ~300 km in the Philippines. Pelagic larval durations (PLD) of 15-45 days offer different survival probabilities which obviously depend on the regional oceanography. Especially the seasonal ocean currents and circulatory systems [12] during the summer monsoon seem suitable to facilitate transport to and from the Socotra Archipelago for species at the longer end of the PLD range. Physical connectivity simulations [96] can provide the templates for potential dispersal patterns. At least for reef-associated species the picture is complicated by the antagonistic effects of the monsoon-related cold upwelling. They are limiting both, the distribution of biogenic reefs and the survivability of fish larvae, not least in relation to life history strategies and ecological spezialisation of individual species [103]. A rigorous study approach tracing the aforementioned studies using different individual-based dispersal models and population genetics, applied to the main seasonal circulatory features, in considering a disjunct availability of critical habitats, could elucidate spatio-temporal patterns of larval connectivity and their implications to the biogeography of the wider south-eastern Arabian region.

Biogeographic studies of other marine taxa from the Socotra Archipelago provide instructive comparative insights. According to recent multi-taxon studies [13, 30] a boundary triggered by the main upwelling areas at the intersection between the Gulf of Aden and the Arabian Sea (compare Bellwood and Wainwright 2002, Fig 5e) [67] rather acts as partly permeable ecological filter than as physical barrier to dispersal (see [104]). The present study rather argues that this filter has two “fronts”, an eastern one acting towards the central Indian Ocean, and a western one within the Gulf of Aden (besides the sill of Bab al-Mandeb). The hypothesised permeability of these filters is supported by DiBattista et al. (2017) [32] who found no genetic partitioning in nine out of 11 species of reef fishes in the seas surrounding Socotra. Reef-building corals of the Socotra Archipelago show strong zoogeographic affinities to those of the Arabian Seas and the Western Indian Ocean, whilst “forming a unique composite fauna from these and other faunal provinces including rare species with restricted and/or highly disjunct global distributions” [11] (see also [12]). Veron et al. (2015) [65] include a regional cluster analysis for the western Indian Ocean and the Red Sea, arguing that the Socotra Archipelago has closer affinities to east Somalia and east Africa, than to the Gulf of Aden and the Red Sea. The macroalgae assemblages of the Socotra Archipelago display a high biogeographical affinity to East Africa, whilst at the same time including many species, which are characteristic of Arabia. Affinities with distant regions and disjunctly distributed taxa were also prominent, with the Archipelago functioning as a stepping stone for the dispersal of species. (i.e., [18, 19, 100, 105]). In conclusion, largely consistent biogeographic patterns obviously exist for the assemblages of at least three major marine taxa (macroalgae, corals and fishes). The proposed alternative ecoregional delineations appear to well represent these joint patterns and the corresponding high levels of fish diversity [4], coral diversity [11, 65], seaweed and seagrass diversity [100, 106, 107] and overall marine biodiversity [108, 109], compared to other Arabian ecoregions (e.g. [13]).

The Archipelago is situated in dynamic regional environment with a complex evolutionary history. Hybrids represent a characteristic element of the fish fauna of the Socotra Archipelago (i.e., [4, 29]). DiBattista et al. (2015) [29] (compare also [110]) recognized the Archipelago as a main hotspot for fish hybridization globally and as “suture zone” *sensu* Remington (1968) [111]. Kemp (1998) [5] had already noticed a high frequency of sympatric occurrences of “Indian Ocean and Arabian sister species” in the Socotra Archipelago. Studying the phylogeography of the angelfish genus *Pomacanthus* Hodge et al. (2013) [28] stated that the Archipelago is part of the area with the highest co-occurrence of sister species in the Western and Central Indian Ocean. This underscores the role of Socotra as a suture or transition zone (also “crossroads” *sensu* DeVantier et al. 2004) [11] and offers an explanation for the extraordinarily high numbers of hybrids recorded. It also suggests that con-specific populations genetically still mix (incomplete lineage sorting) and sister species again mix (hybridization, introgression), respectively, around the archipelago that elsewhere segregate (compare [80]; but see [33] for an exception to this assumption in three species of chocolate-dipped damselfish, genus *Chromis*).

According to Borsa et al. (2015 [83]; also [34, 112, 113]) phylogeographic subdivisions generally corroborate biogeographic provinces inferred from species distributions (“checklists” *sensu* e.g. Kulbicki et al. (2013) [40]). A notable exception is the Indo-Pacific boundary (Briggs and Bowen 2012) [39]. At this boundary, the Christmas and Cocos (Keeling) Islands have been revealed as a prominent suture zone (e.g., [114–116]) and compared to the Socotra suture zone ([33, 117]). Crandall et al. (2019) [118], however, found low support to the partitioning of the Indo-Pacific Ecoregions and Provinces of Spalding et al. (2007) [38] based on fish phylogeography. How to best capture suture and transition zones in ecoregional schemes, and how to reconcile phylogeographic evidence with them, including the role of endemism [22, 38, 39, 71, 113], may further fuel research on the ecoregional working hypotheses presented in this study.

## Conclusions

The present study elucidates the position of the Socotra Archipelago in terms of distributional ichthyogeography, updating Kemp (1998) [5]. Additional critical inferences are made with regard to the ichthyogeographic and ecoregional subdivision of the Arabian region, suggesting amendments to the classification schemes of Briggs and Bowen (2012) [39], Kulbicki et al. (2013) [40] and especially the ‘Marine Ecoregions of the World’ (MEOW) of Spalding et al. (2007) [38]. The results of the resemblance analyses of the circum-Arabian ecoregions reveal:

- The Socotra Archipelago has close affinities to a putative ecoregion combining the eastern Gulf of Aden and southern Oman.
- The Socotra Archipelago is more closely related to the Arabian Sea coast of Oman (southern Oman and central Oman) than to ecoregions in the Red Sea and a putative ecoregion in the western Gulf of Aden.
- The Gulf of Aden does not represent a consistent ecoregion, because its eastern and western parts are less close to one another than to other ecoregions.
- The Socotra Archipelago and the eastern Gulf of Aden should not be assigned to the same province as the Red Sea ecoregions.
- The coastal fish faunas of the southern Red Sea and the western Gulf of Aden have close affinities with each other across the Strait of Bab al-Mandeb.
- The Arabian/Persian Gulf is least related to all other Arabian ecoregions studied here.

The authors conclude that recognising the Socotra Archipelago as a distinct ecoregion, either on its own or in combination with affiliated mainland areas, best reflects the ichthyogeographic data. It also adequately recognizes its high levels of fish diversity and overall marine biodiversity, as compared to other Arabian ecoregions. The study thus proposes two alternative ecoregional delineations to the MEOW, serving as working hypotheses for onward research.

## Supporting information

S1 Data Distributional checklist according to 10 putative ecoregions. Matrices (a) listing 1292 species of twenty key families, including eight key families, and (b) counting them by family, according to their presence in ten putative circum-Arabian ecoregions and Kenya (898 species), including additional 11 countries and island groups of the Western Indian Ocean.
(XLSX)

S2 Data ANOSIM results of province-level combinations testing. List of the ANOSIM results of the statistical validation of 28 plausible *a priori* “province-level” designations (combinations) of the 10 Arabian ecoregional units (and Kenya), including pairwise test results.
(XLSX)

S3 Data SIMPROF and Kruskal Stress values of core cluster and nMDS analyses. List of the SIMPROF significance values of within-cluster data structure and of nMDS stress values pertinent to core analyses illustrated in the Figs 3a, 3b, 4a, 4b, 5, 6, 8a and 8b.
(XLSX)

S4 Data Species distributions. List of 234 species of eight key families from Socotra Archipelago, classified according to a system of 12 global species distribution range patterns (based on Zajonz et al. 2019).
(XLSX)

S1 Fig Dendrograms of additional hierarchical agglomerative cluster analyses of subregional resemblance patterns. Analyses of incidence-based resemblance patterns according to the dataset underlying Fig 3, restricted to (a) species (65) of four families only (Chaetodontidae, Pomacanthidae, Acanthuridae, Balistidae) used in the analysis by Kemp (1998); and, (b) adding Pomacentridae according to Kemp (2000) (103 spp.). See Fig 2 for locations; superposed with symbols representing *a priori* basin designations.
(DOCX)

S2 Fig Individual resemblance patterns of eight key families in 10 putative Arabian ecoregions. Dendrograms of hierarchical agglomerative cluster analyses based on Hellinger’s distance, complementing Fig 4, representing (a) Acanthuridae, (b) Balistidae, (c) Chaetodontidae, (d) Pomacanthidae, (e) Pomacentridae, (f) Labridae, (g) Pseudochromidae, and (h) Serranidae; superposed with symbols representing the statistically (ANOSIM) most valid *a priori* Combination U of province-level designations.
(DOCX)

S3 Fig nMDS plot corresponding to Fig 8a and 8b. Non-metric Multi-dimensional Scaling plot based on the Hellinger’s distance matrix underlying the hierarchical agglomerative cluster analyses of 10 putative Arabian ecoregions, and 12 countries and island groups of the wider Western Indian Ocean (Fig 8a, eight key families, 604 species; Fig 8b, twenty families, 1292 species); with (a) superposed with symbols representing the statistically (ANOSIM) most valid *a priori* Combination U of province-level designations.
(DOCX)

S4 Fig Additional charts. Supplementary figures and explorative analyses serving to validate results.
(DOCX)
