# Supplementary material for: Biogeography of the coastal fishes of the Socotra Archipelago: Challenging current ecoregional concepts
Source: PLoS One. 2022 Apr 29;17(4):e0267086. doi: 10.1371/journal.pone.0267086 (PMC9053782; doi:10.1371/journal.pone.0267086)

**Zajonz, U., Lavergne, E., Bogorodsky, S.V. & Krupp, F.** Biogeography of the Coastal Fishes of the Socotra Archipelago: Challenging Current Ecoregional Concepts. PLoS ONE (2022 acc.) **– Supporting Information –**

**S1 Fig. Dendrograms of additional hierarchical agglomerative cluster analyses of subregional resemblance patterns.** Analyses of incidence-based resemblance patterns according to the dataset underlying Fig 3, restricted to (a) species (65) of four families only (Chaetodontidae, Pomacanthidae, Acanthuridae, Balistidae) used in the analysis by Kemp (1998); and, (b) adding Pomacentridae according to Kemp (2000) (103 spp.). See Fig. 2 for locations; superposed with symbols representing *a priori* basin designations.


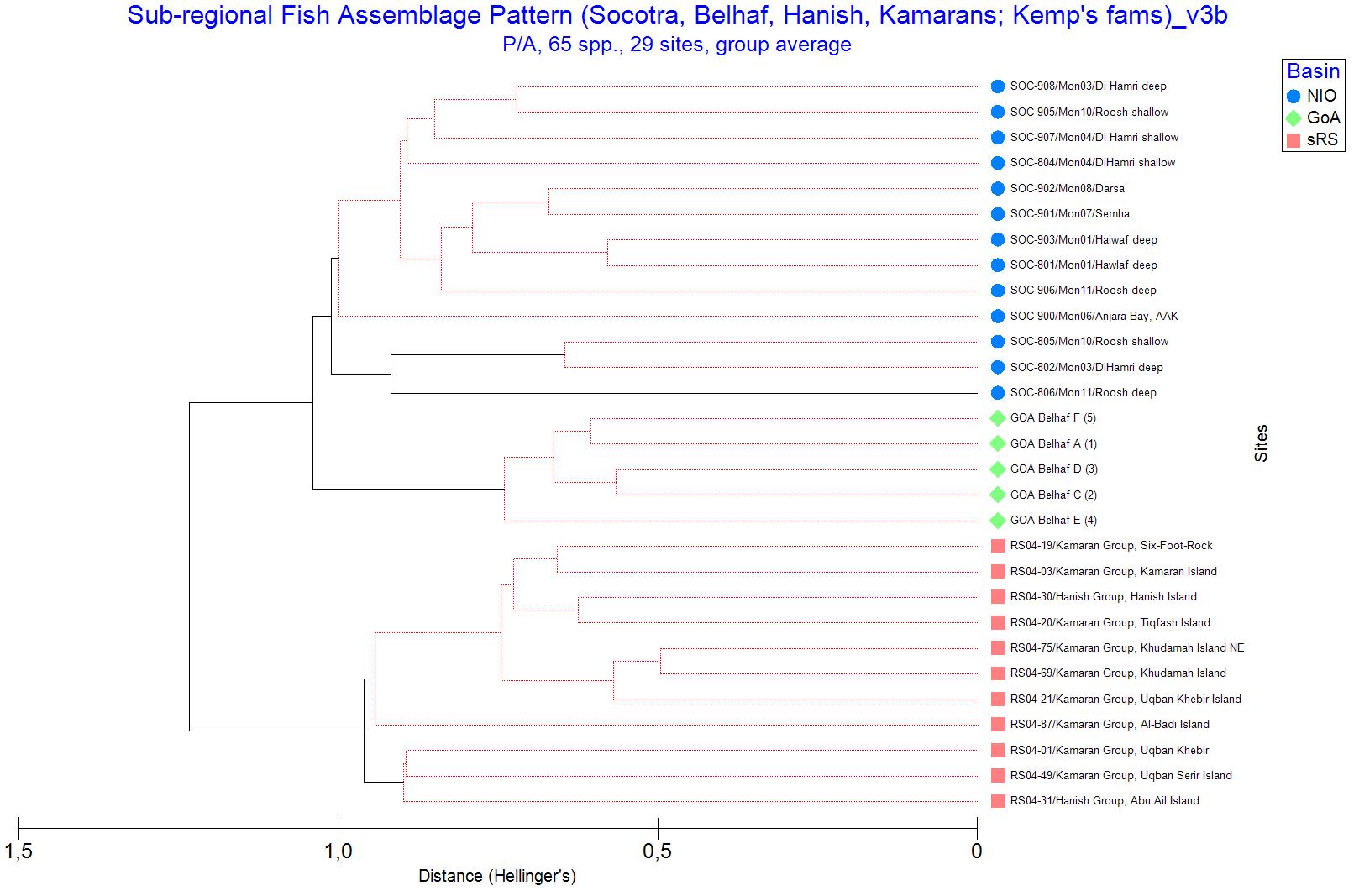


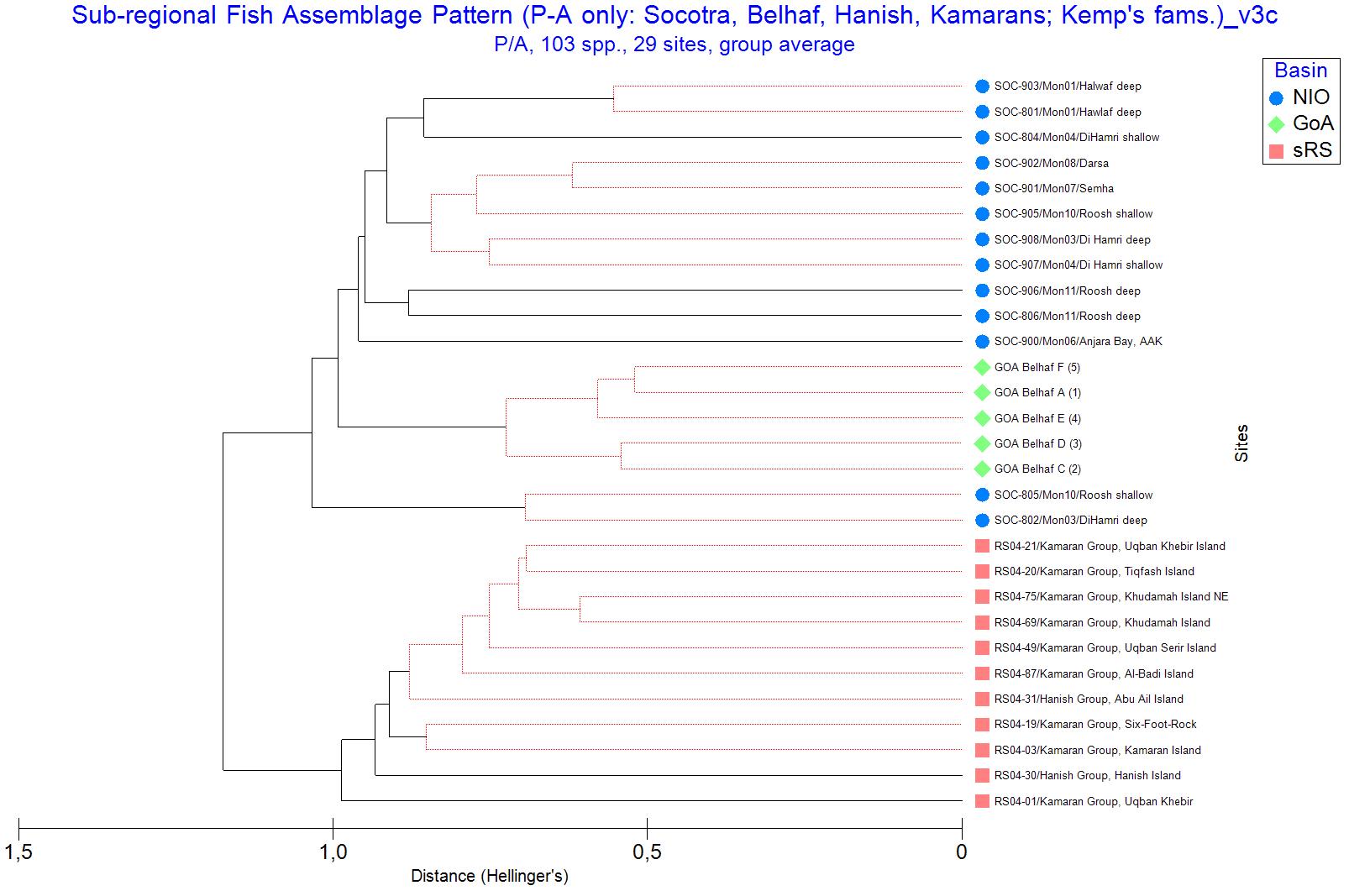

Supplement: S1 Fig — Analyses of incidence-based resemblance patterns according to the dataset underlying Fig 3, restricted to (a) species (65) of four families only (Chaetodontidae, Pomacanthidae, Acanthuridae, Balistidae) used in the analysis by Kemp (1998); and, (b) adding Pomacentridae according to Kemp (2000) (103 spp.). See Fig 2 for locations; superposed with symbols representing a priori basin designations. (DOCX) [file pone.0267086.s005.docx]
