# Supplementary material for: Biogeography of the coastal fishes of the Socotra Archipelago: Challenging current ecoregional concepts
Source: PLoS One. 2022 Apr 29;17(4):e0267086. doi: 10.1371/journal.pone.0267086 (PMC9053782; doi:10.1371/journal.pone.0267086)

**Zajonz, U., Lavergne, E., Bogorodsky, S.V. & Krupp, F.** Biogeography of the Coastal Fishes of the Socotra Archipelago: Challenging Current Ecoregional Concepts. PLoS ONE (2022 acc.) **– Supporting Information –**

**S2 Fig. Individual resemblance patterns of eight key families in 10 putative Arabian ecoregions.** Dendrograms of hierarchical agglomerative cluster analyses based on Hellinger’s distance, complementing Fig 4, representing (a) Acanthuridae, (b) Balistidae, (c) Chaetodontidae, (d) Pomacanthidae, (e) Pomacentridae, (f) Labridae, (g) Pseudochromidae, and (h) Serranidae; superposed with symbols representing the statistically (ANOSIM) most valid *a priori* Hypothesis U of province-level designations.


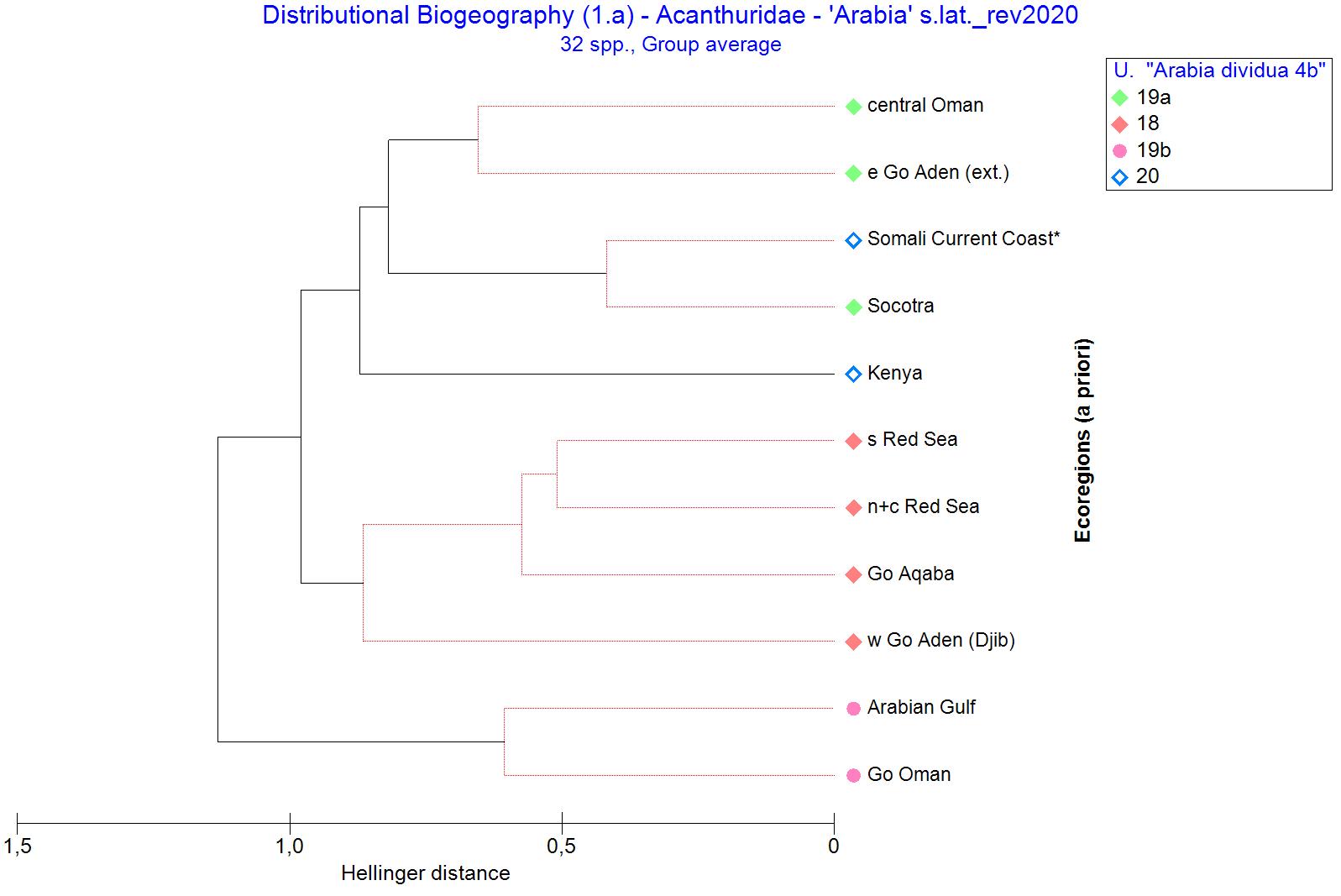


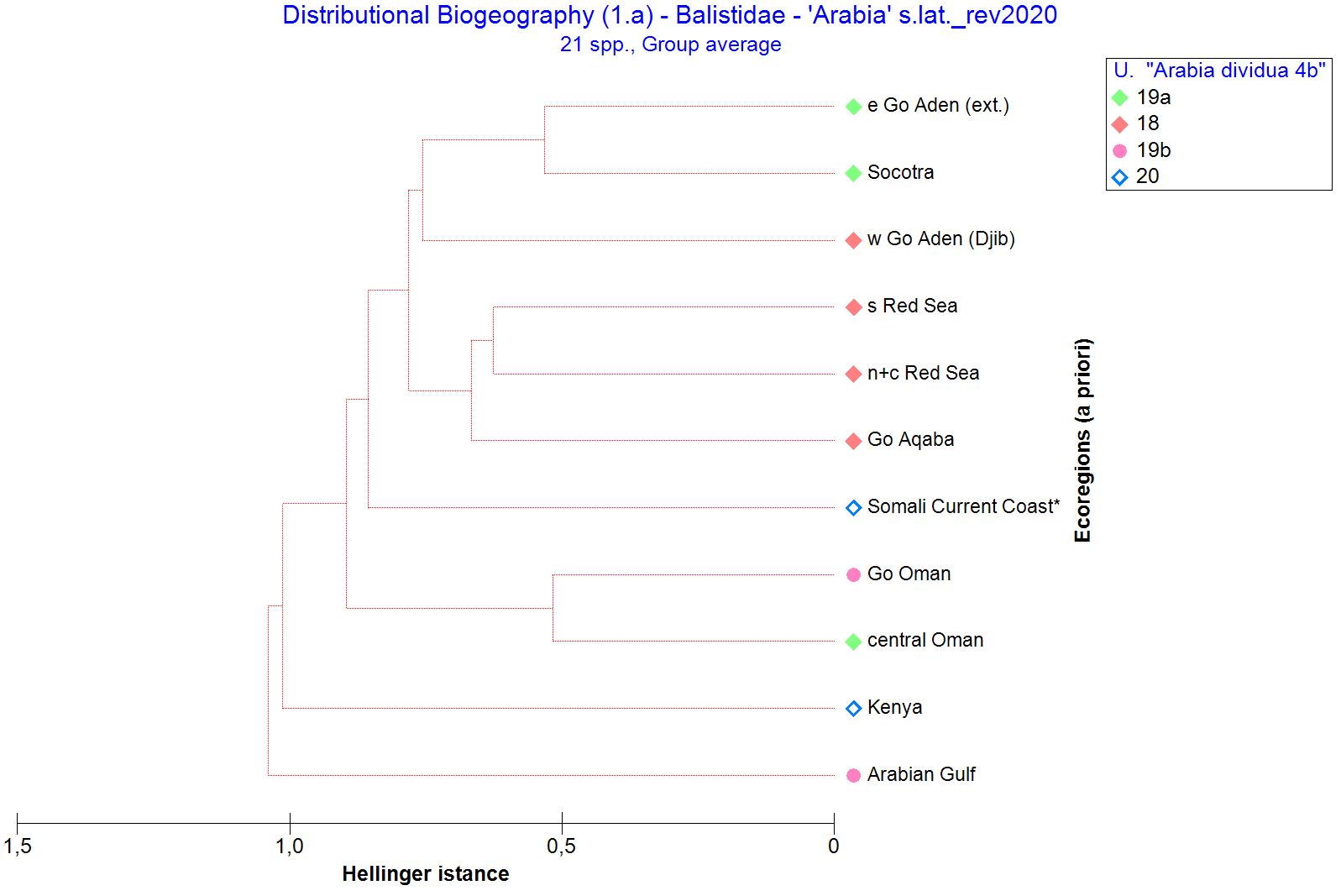


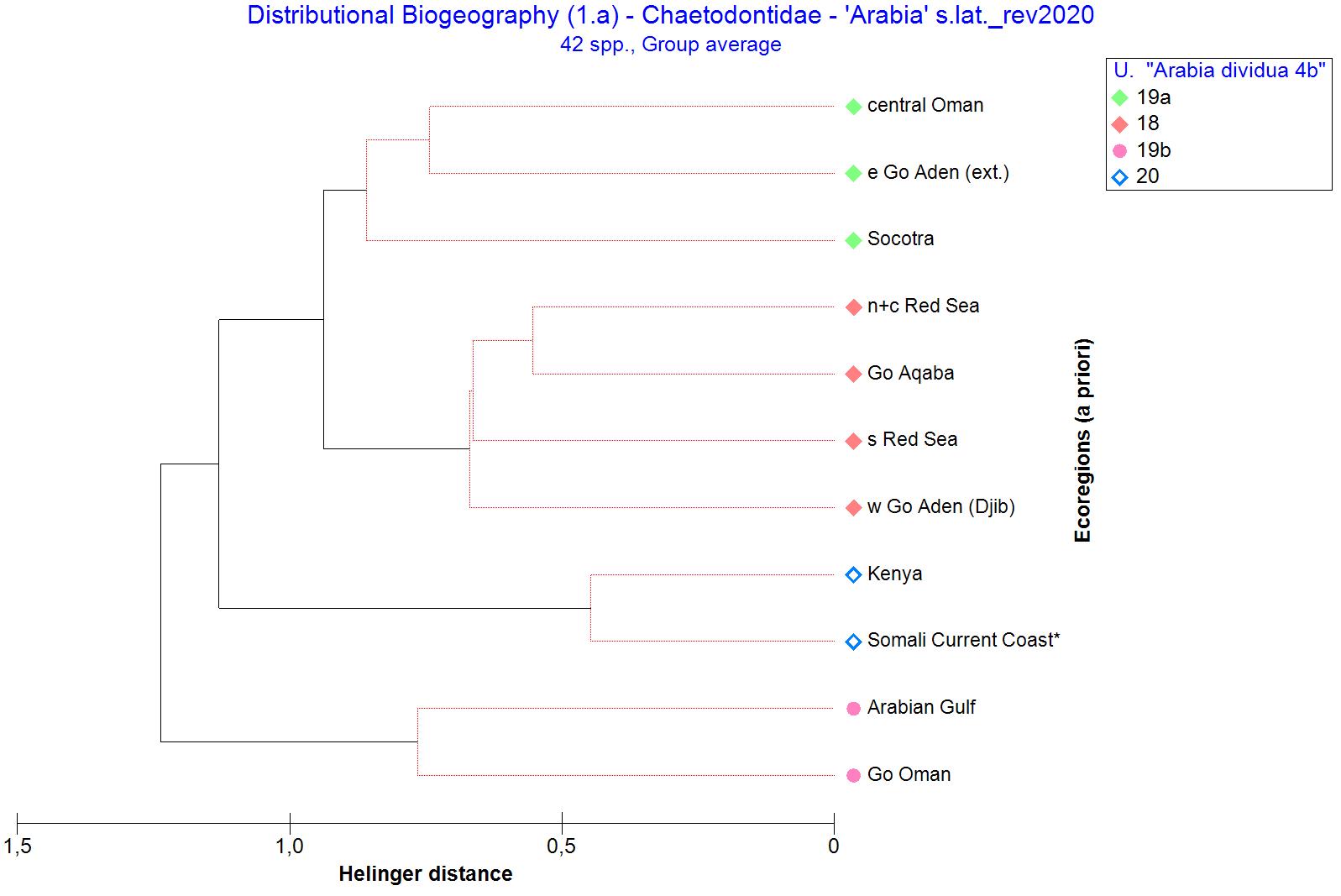


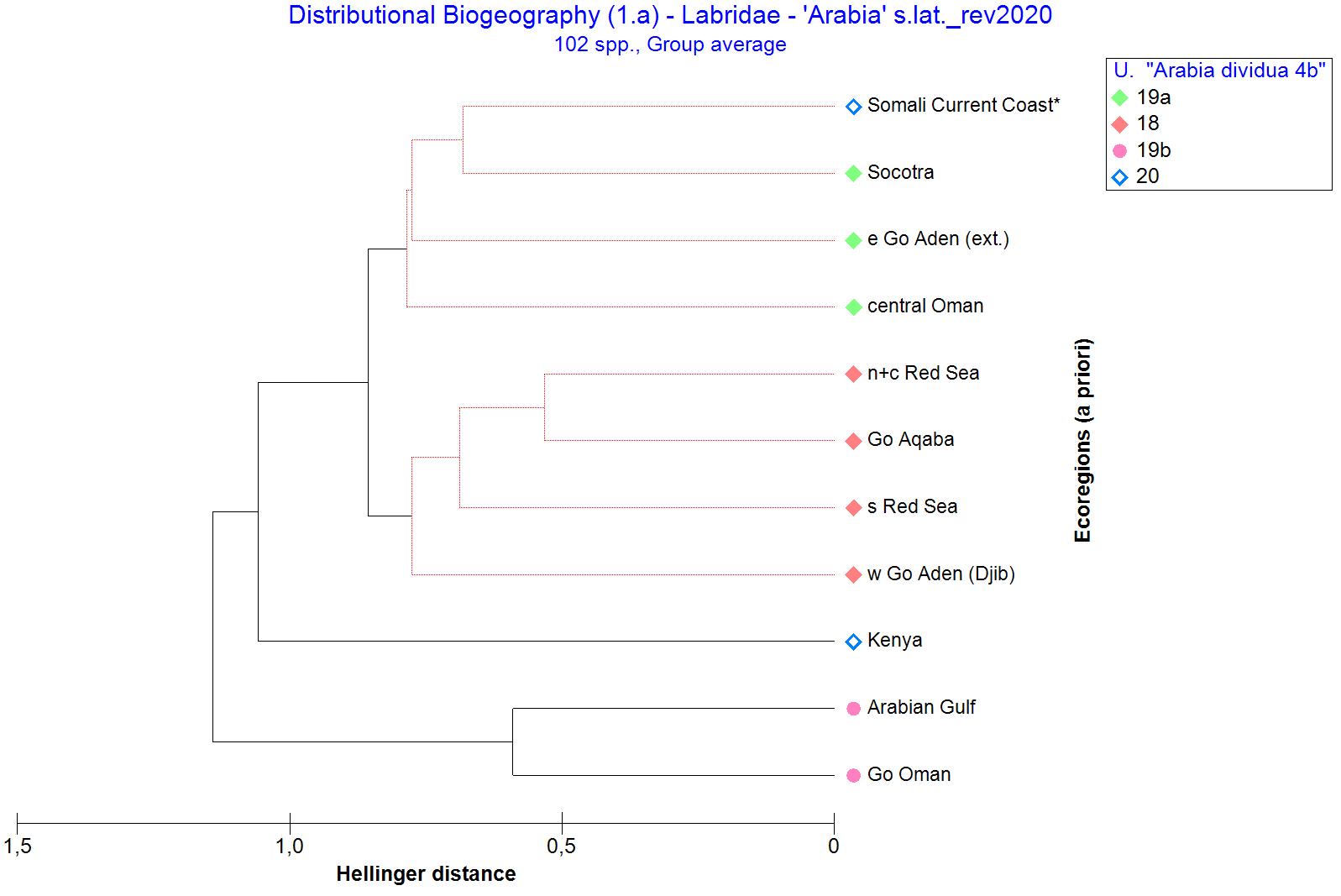


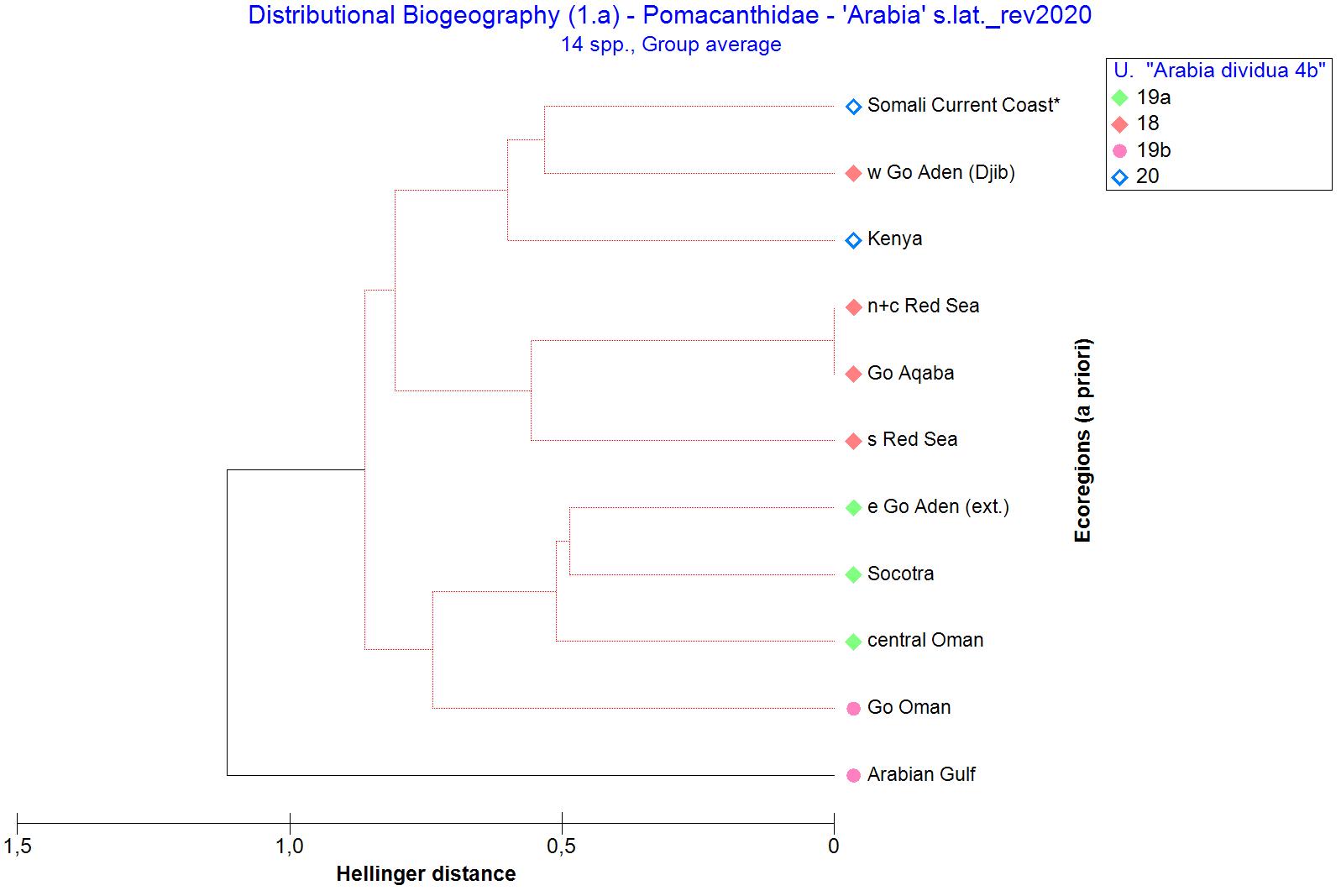


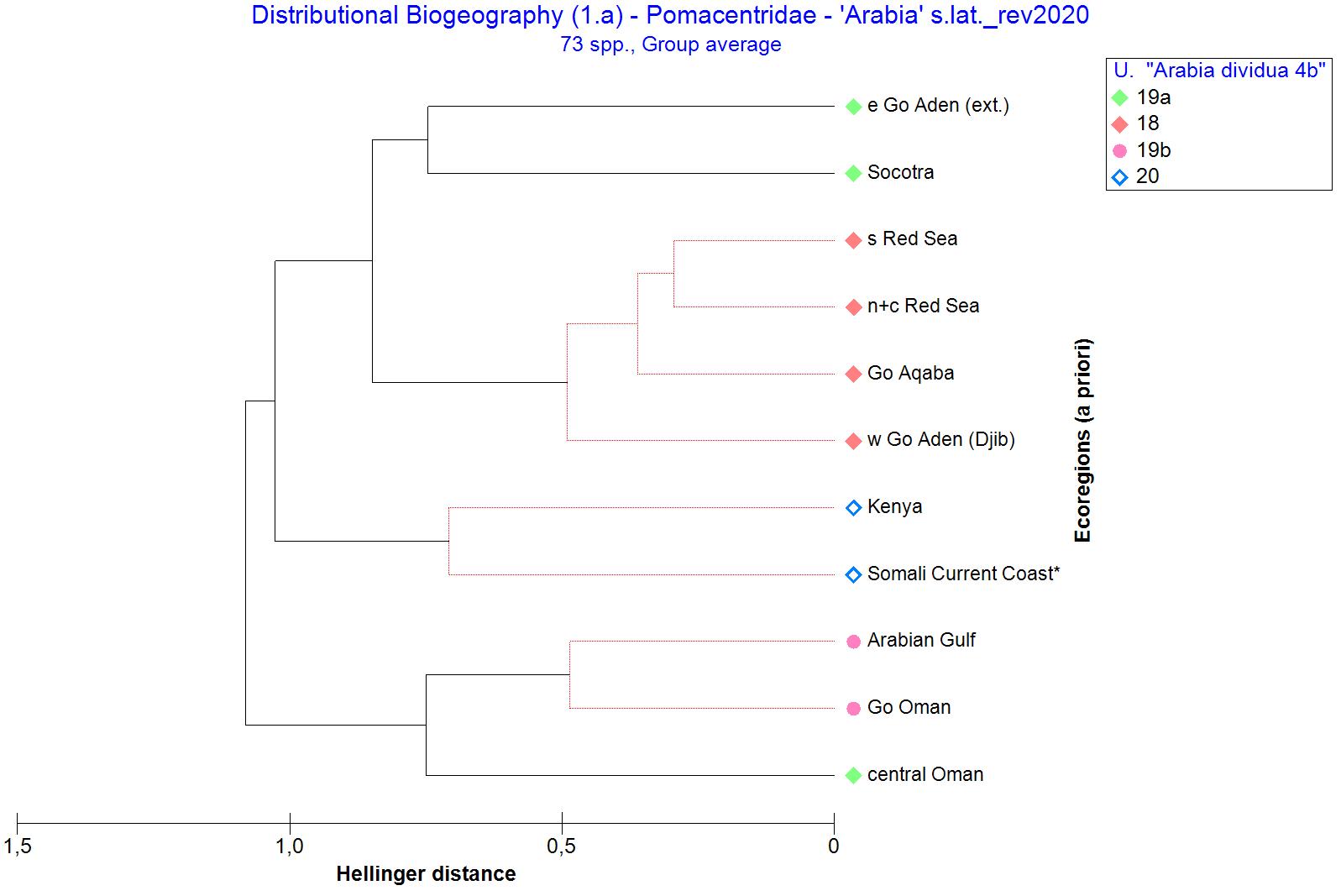


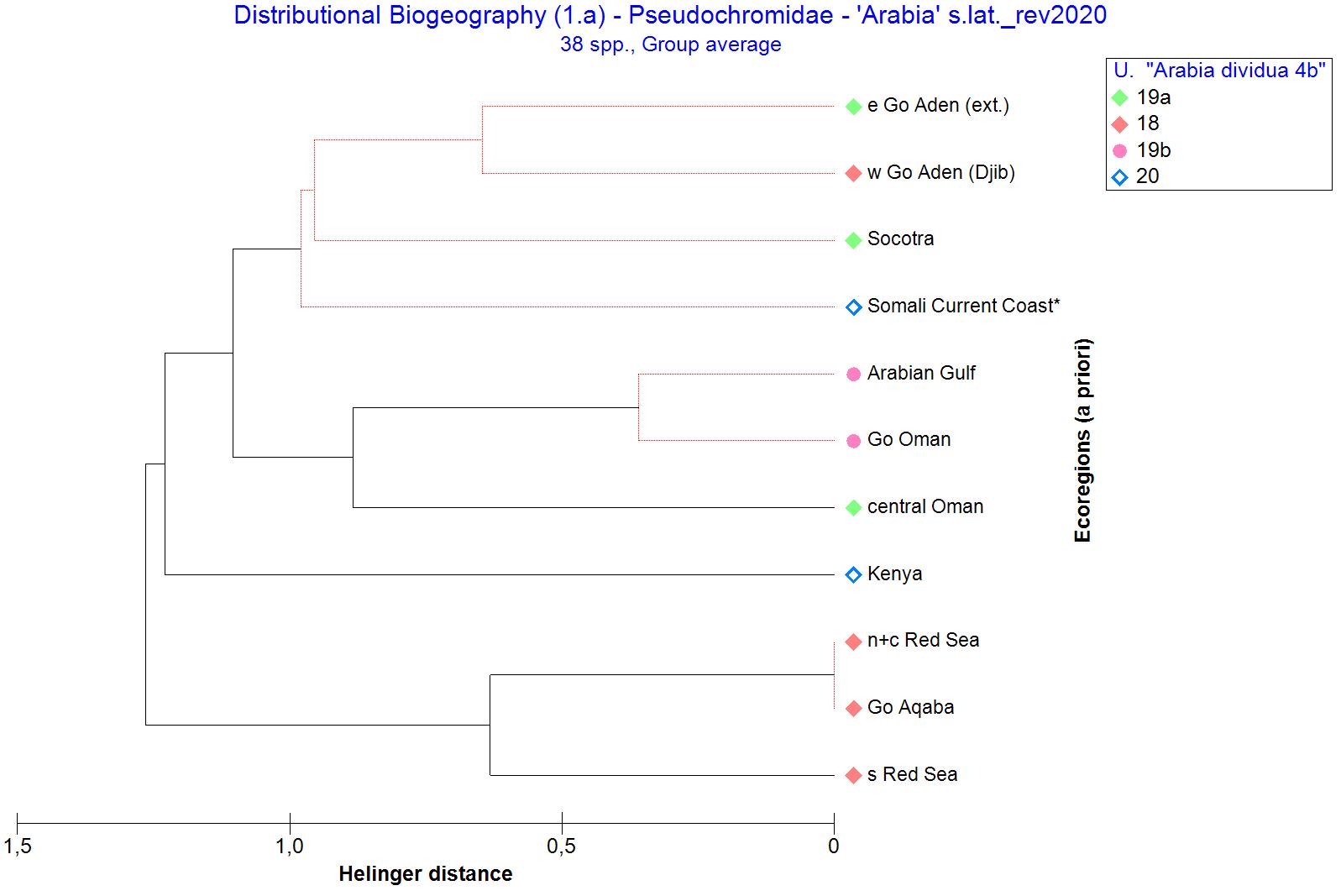


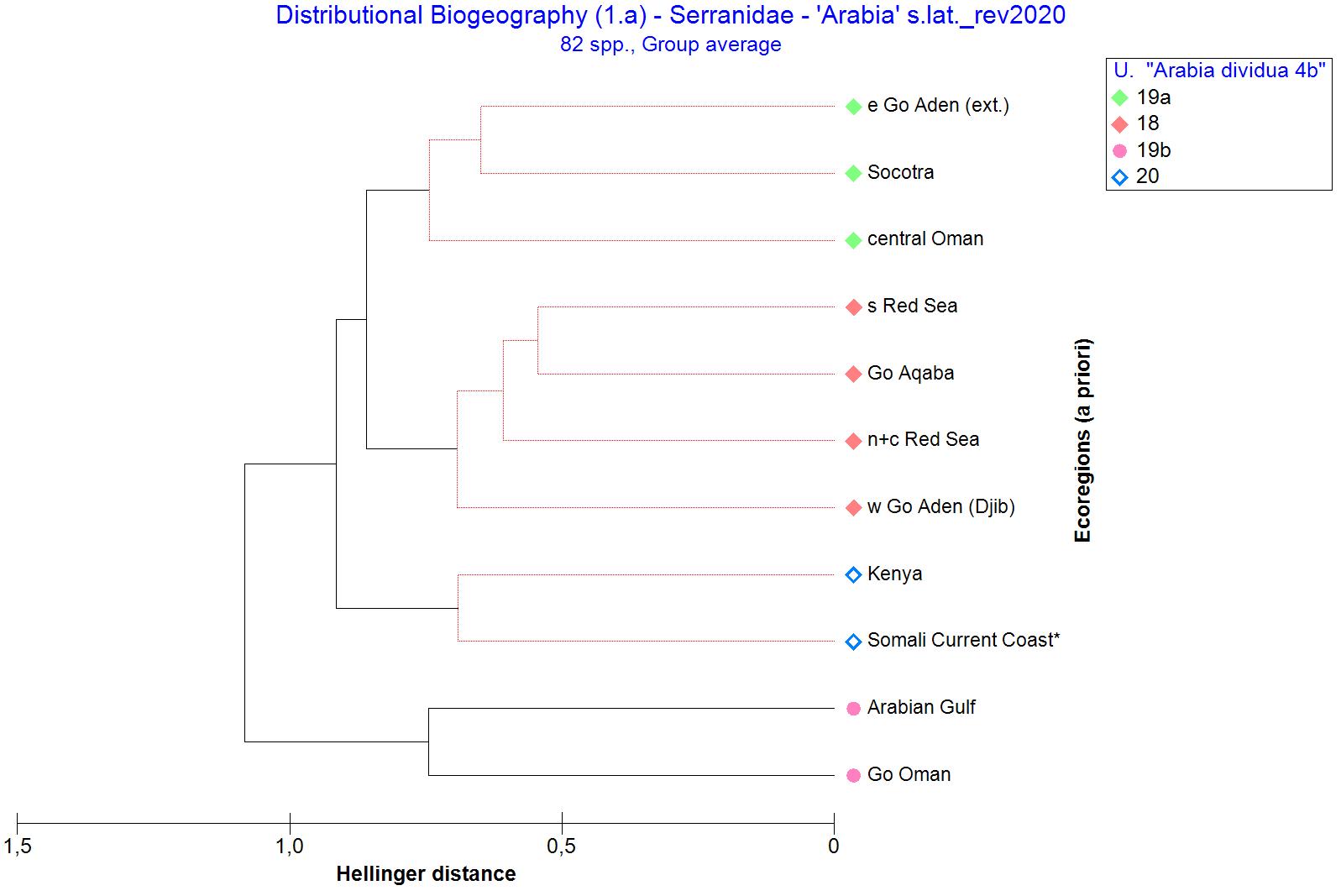

Supplement: S2 Fig — Dendrograms of hierarchical agglomerative cluster analyses based on Hellinger’s distance, complementing Fig 4, representing (a) Acanthuridae, (b) Balistidae, (c) Chaetodontidae, (d) Pomacanthidae, (e) Pomacentridae, (f) Labridae, (g) Pseudochromidae, and (h) Serranidae; superposed with symbols representing the statistically (ANOSIM) most valid a priori Combination U of province-level designations. (DOCX) [file pone.0267086.s006.docx]
