# Supplementary material for: Biogeography of the coastal fishes of the Socotra Archipelago: Challenging current ecoregional concepts
Source: PLoS One. 2022 Apr 29;17(4):e0267086. doi: 10.1371/journal.pone.0267086 (PMC9053782; doi:10.1371/journal.pone.0267086)

**Zajonz, U., Lavergne, E., Bogorodsky, S.V. & Krupp, F.** Biogeography of the Coastal Fishes of the Socotra Archipelago: Challenging Current Ecoregional Concepts. PLoS ONE (2022 acc.) **– Supporting Information –**

**S3 Fig. nMDS plot corresponding to Figs 8 a-b**. Non-metric Multi-dimensional Scaling plot based on the Hellinger’s distance matrix underlying the hierarchical agglomerative cluster analyses of 10 putative Arabian ecoregions, and 12 countries and island groups of the wider Western Indian Ocean (Fig 8 a, eight key families, 604 species; Fig. 8 b, twenty families, 1292 species); with (a) superposed with symbols representing the statistically (ANOSIM) most valid *a priori* Hypothesis U of province-level designations for Arabia s.lat.


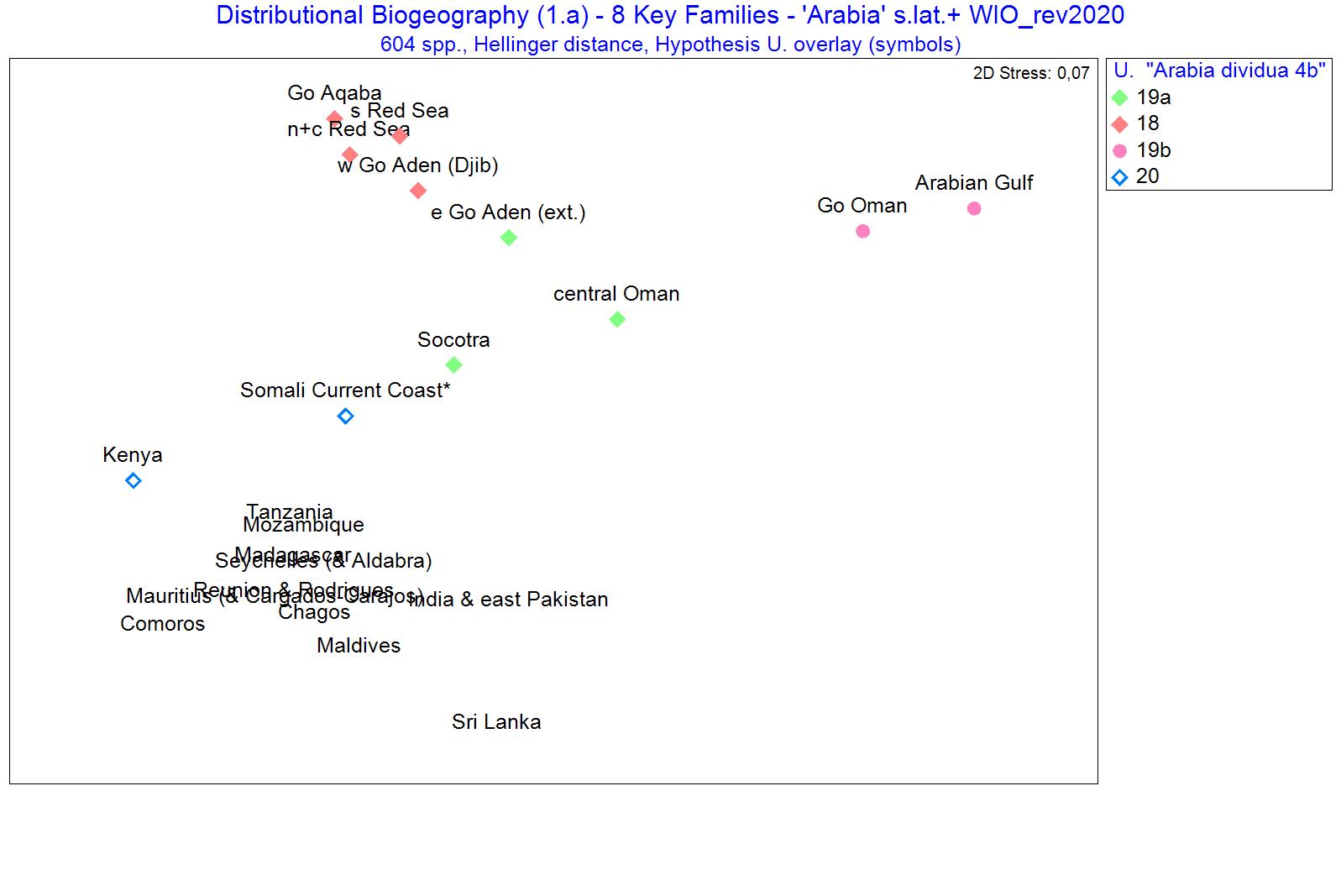


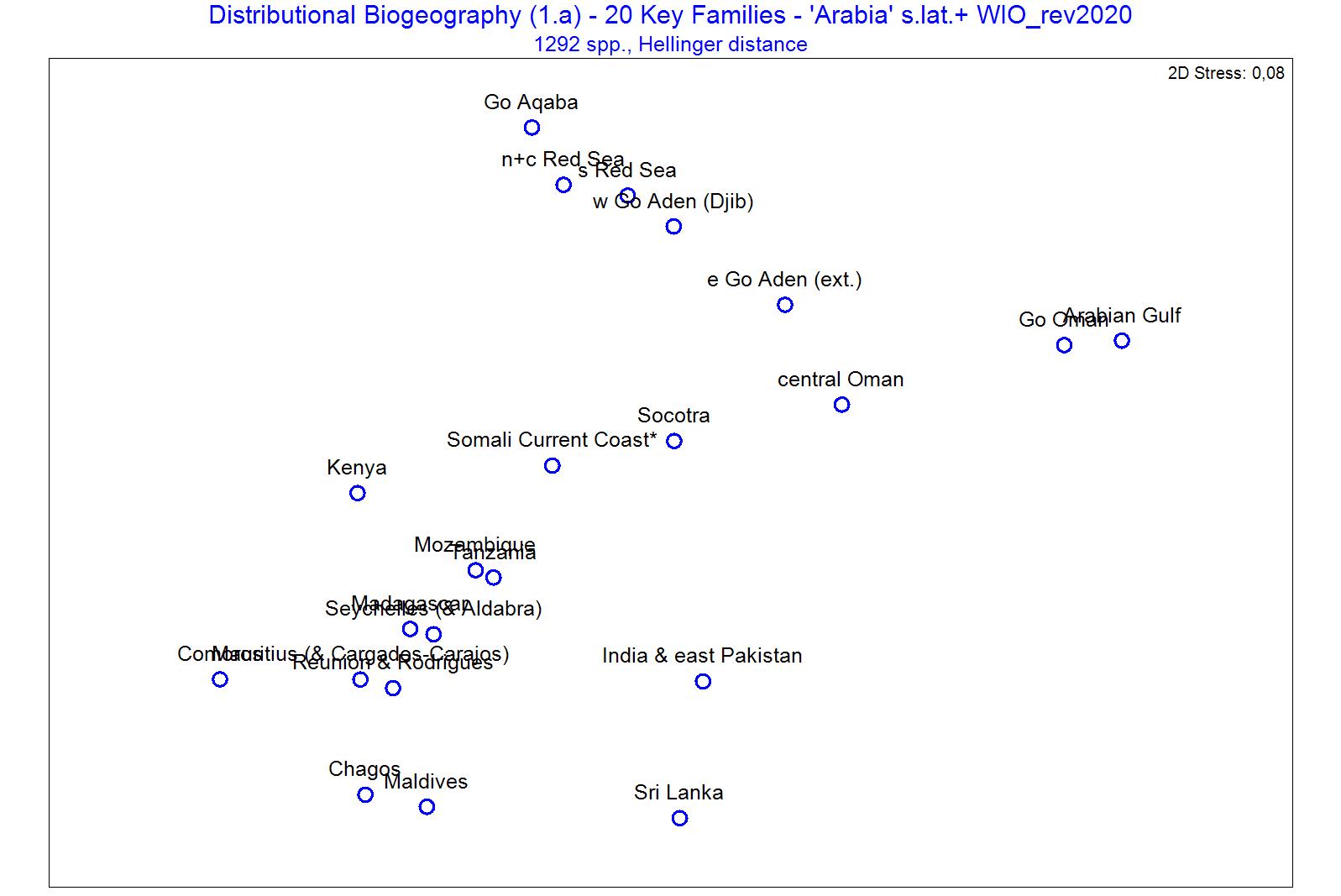

Supplement: S3 Fig — Non-metric Multi-dimensional Scaling plot based on the Hellinger’s distance matrix underlying the hierarchical agglomerative cluster analyses of 10 putative Arabian ecoregions, and 12 countries and island groups of the wider Western Indian Ocean (Fig 8a, eight key families, 604 species; Fig 8b, twenty families, 1292 species); with (a) superposed with symbols representing the statistically (ANOSIM) most valid a priori Combination U of province-level designations. (DOCX) [file pone.0267086.s007.docx]
