# Supplementary material for: Biogeography of the coastal fishes of the Socotra Archipelago: Challenging current ecoregional concepts
Source: PLoS One. 2022 Apr 29;17(4):e0267086. doi: 10.1371/journal.pone.0267086 (PMC9053782; doi:10.1371/journal.pone.0267086)

**Zajonz, U., Lavergne, E., Bogorodsky, S.V. & Krupp, F.** Biogeography of the Coastal Fishes of the Socotra Archipelago: Challenging Current Ecoregional Concepts. PLoS ONE (2022 acc.) **– Supporting Information –**

**S4 Fig. Additional charts.** Supplementary figures and explorative analyses.


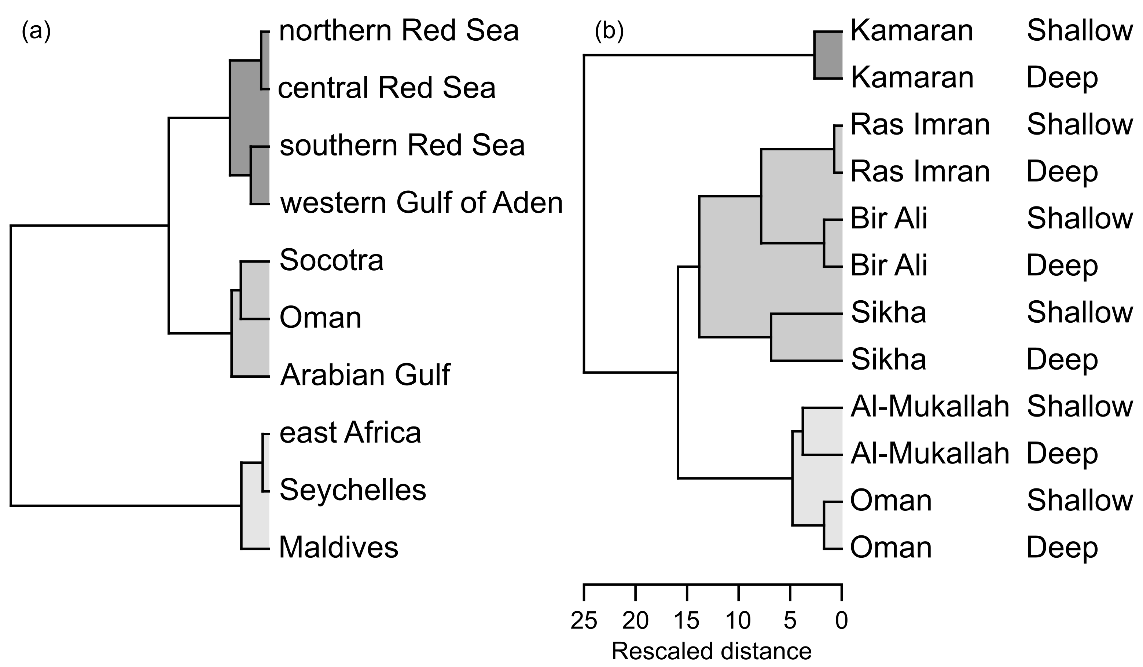


**Kemp’s resemblance patterns of southern Arabian fish assemblages** representing 4 (a) and 5 (b) families, illustrated as dendrogram plots of hierarchical agglomerative cluster analyses (Ward’s method) according to (a) Kemp (1998) based on his own data from the Socotra Archipelago and published and unpublished data from selected surrounding regions; and (b) Kemp (2000b) based on his own data of 26 fish assemblage samples from southern Arabia, aggregated as presence-absence data in six main survey areas at two depth levels.


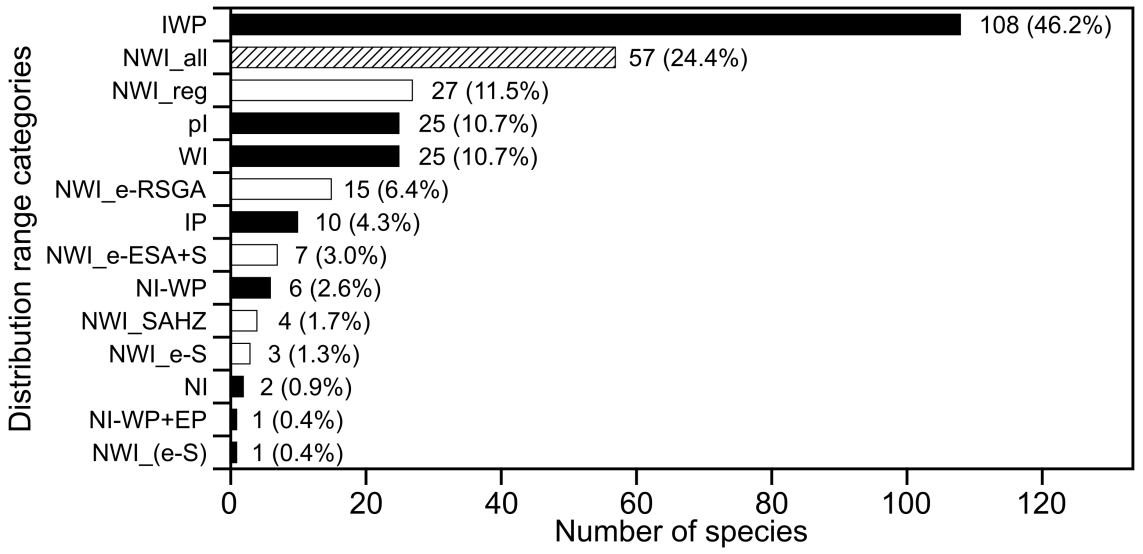


**Frequencies of *a priori* defined global distribution ranges** of 234 species in 8 selected families from Socotra Archipelago, showing numeric frequencies of all distribution range categories and relative proportions of the main range types (based on Zajonz et al*.* 2019); compare with S4 Data for the biogeographical classification of individual species). For the category North-western Indian Ocean (NWI_all, hatched bar) subcategories within this area are also shown (white bars), with their frequencies summing up to the total of NWI_all. Abbreviations: IWP, tropical Indo-West Pacific / NWI_all, North-western Indian Ocean / NWI_reg, North-western Indian Ocean (regular) / pI, pan-tropical Indian Ocean / WI, tropical Western Indian Ocean / NWI_e-RSGA, endemic species of the Red Sea and Gulf of Aden / IP, tropical Indo-Pacific / NWI_e-ESA+S, endemic species of eastern and southern Arabia and Socotra / NI-WP, Northern Indian Ocean to Western Pacific / NWI_SAHZ, southern Arabian Sea hybrid zone / NWI_e-S, endemic species of Socotra / NI, Northern Indian Ocean / NI-WP+EP, Northern Indian Ocean to Western Pacific and Eastern Pacific.


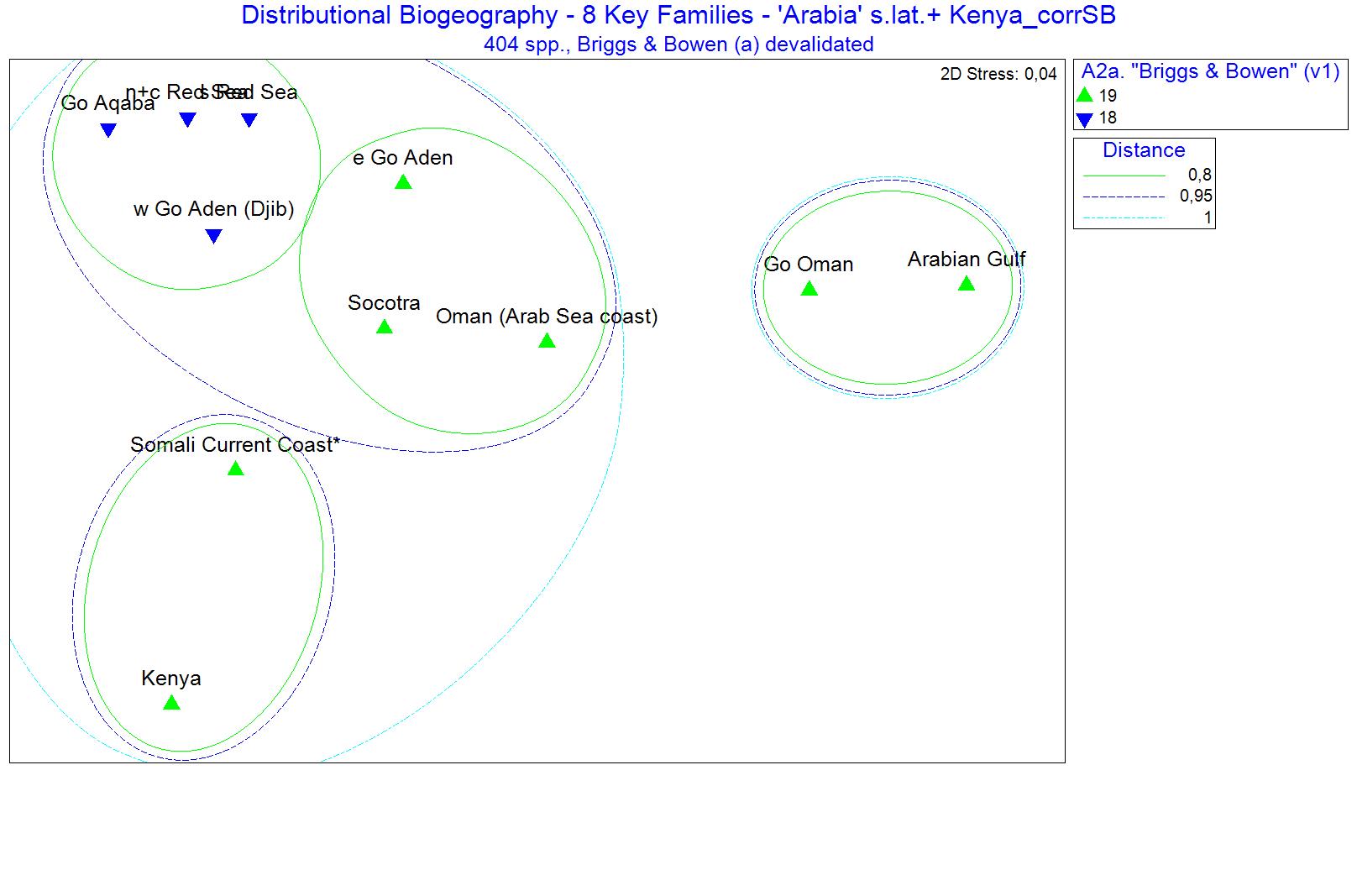


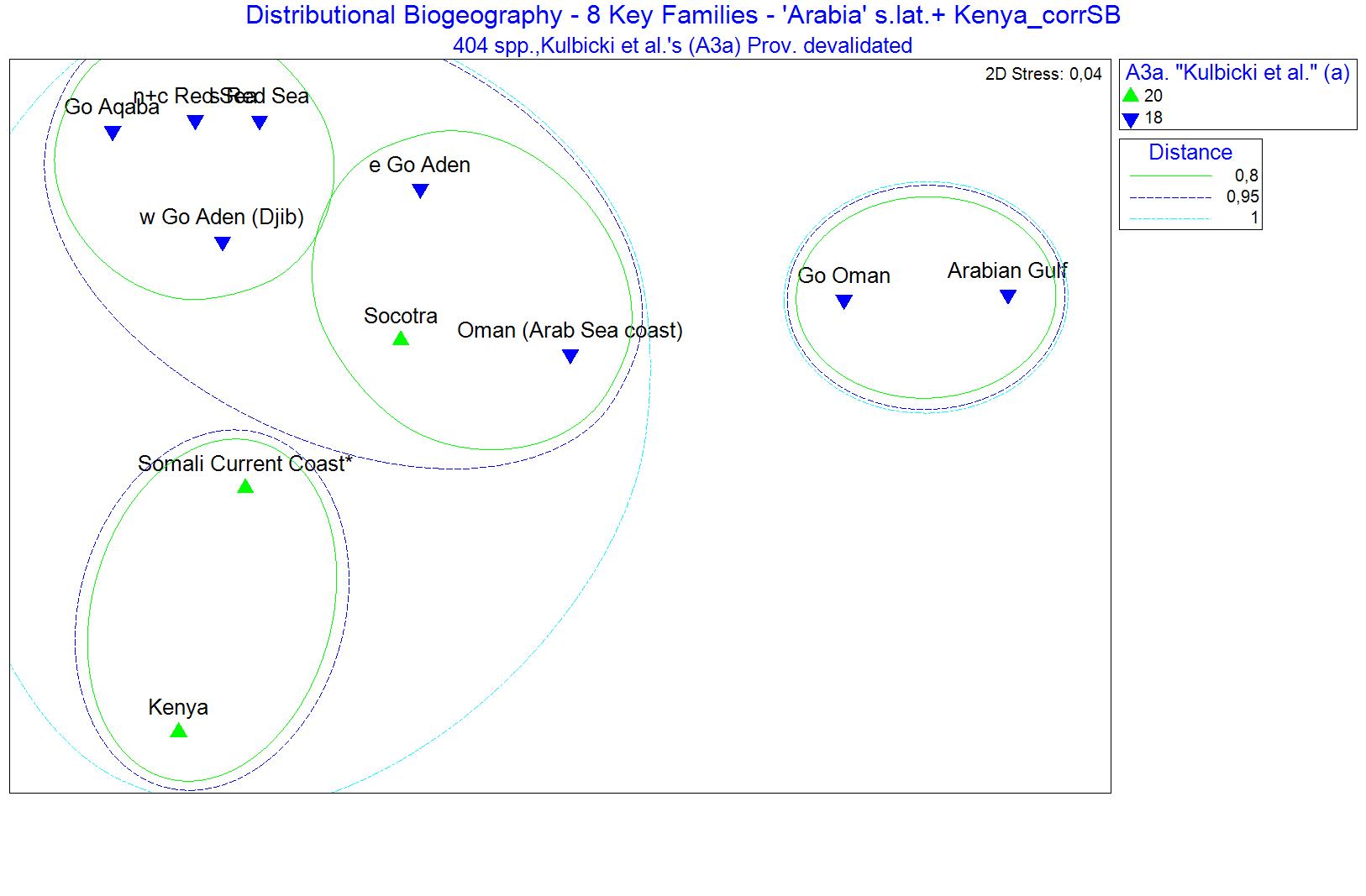


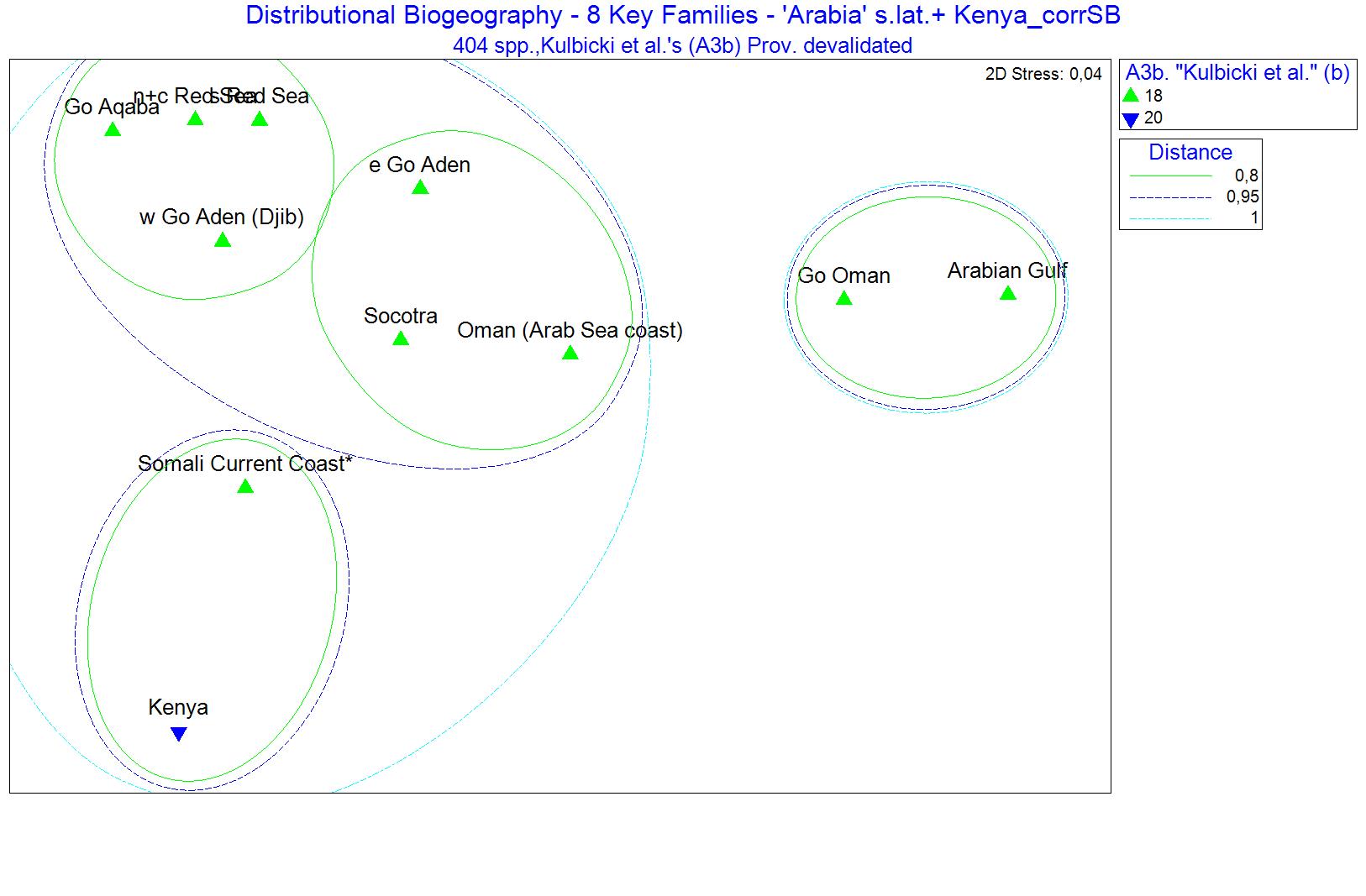


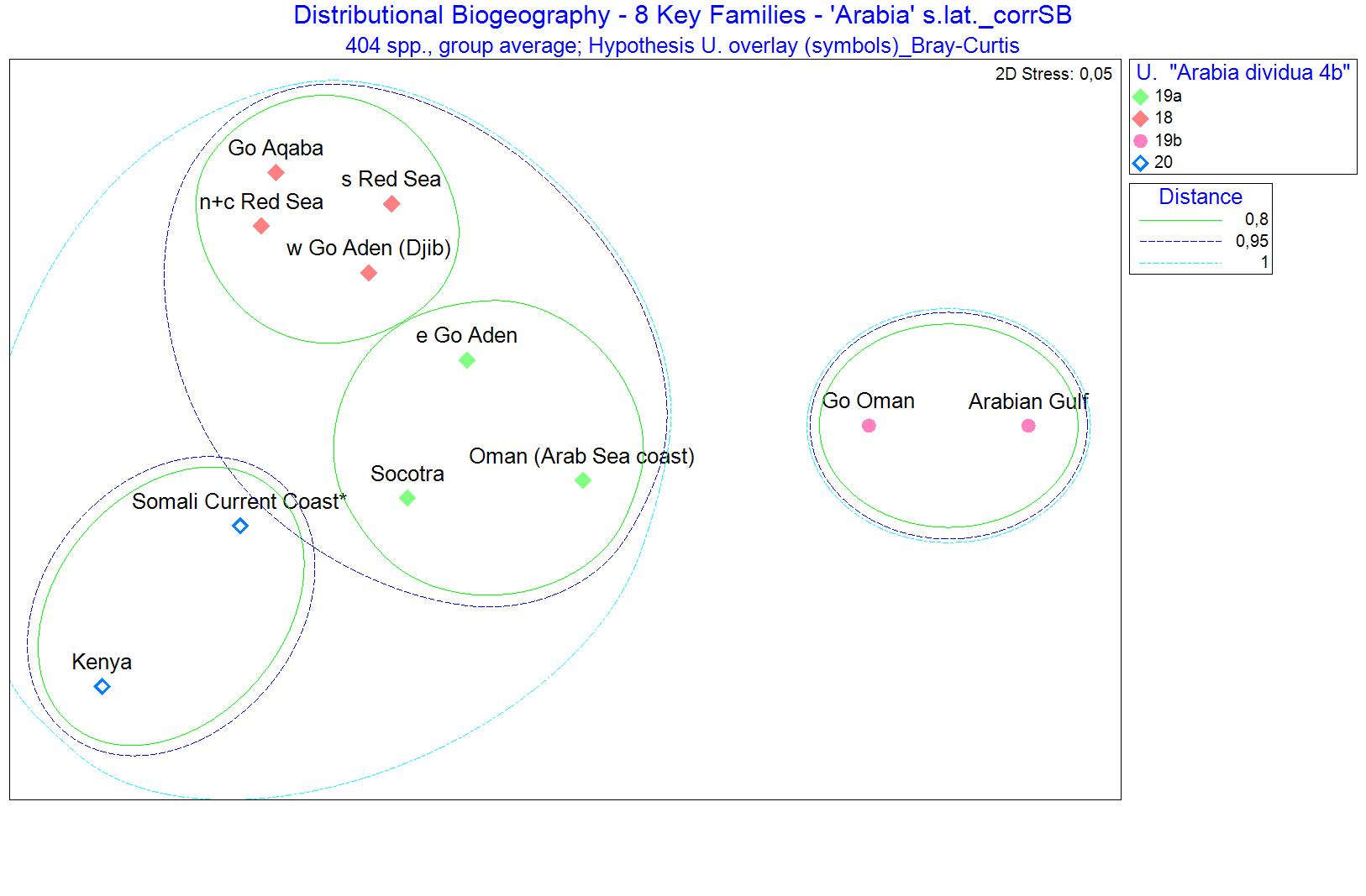


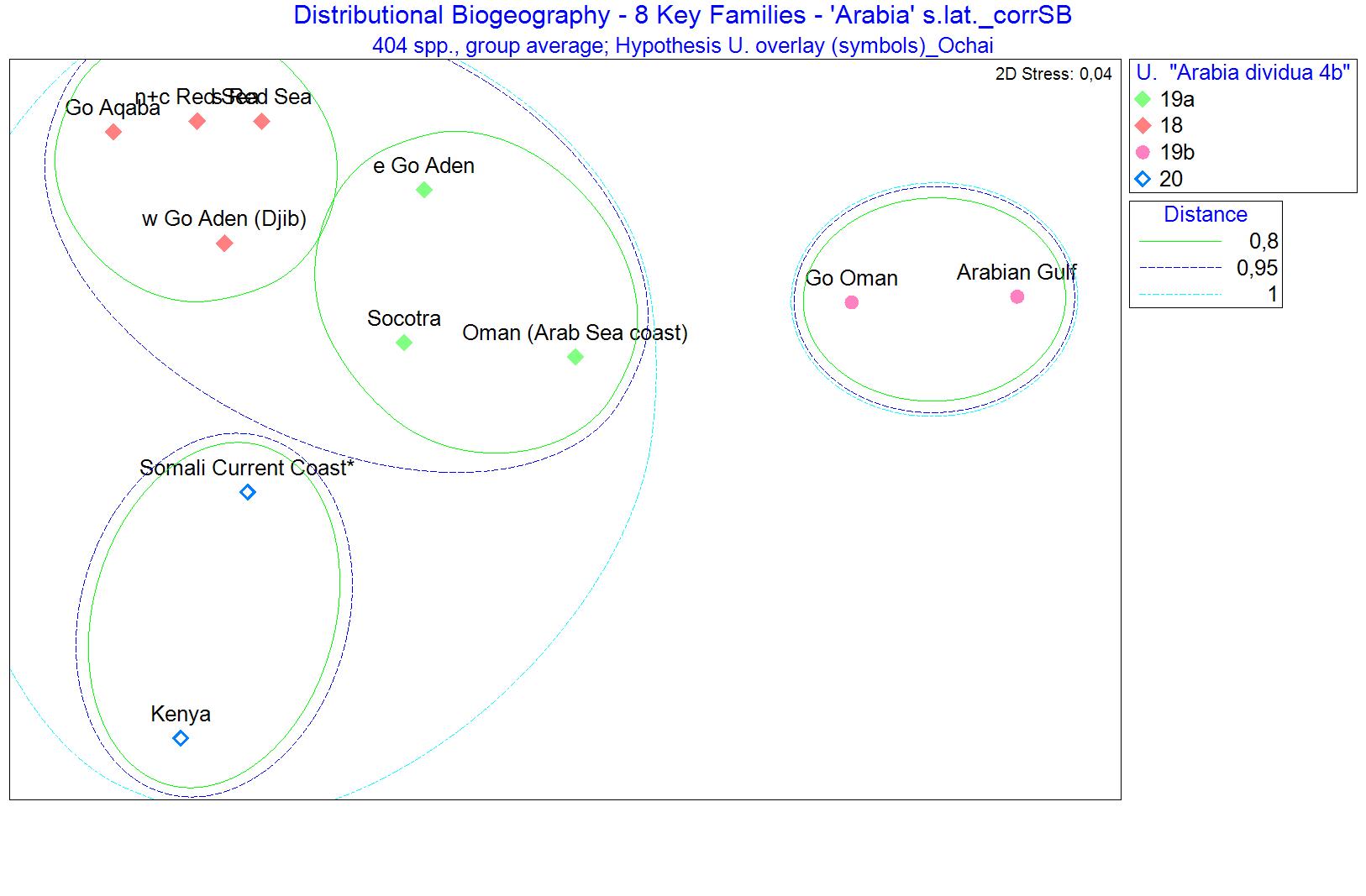


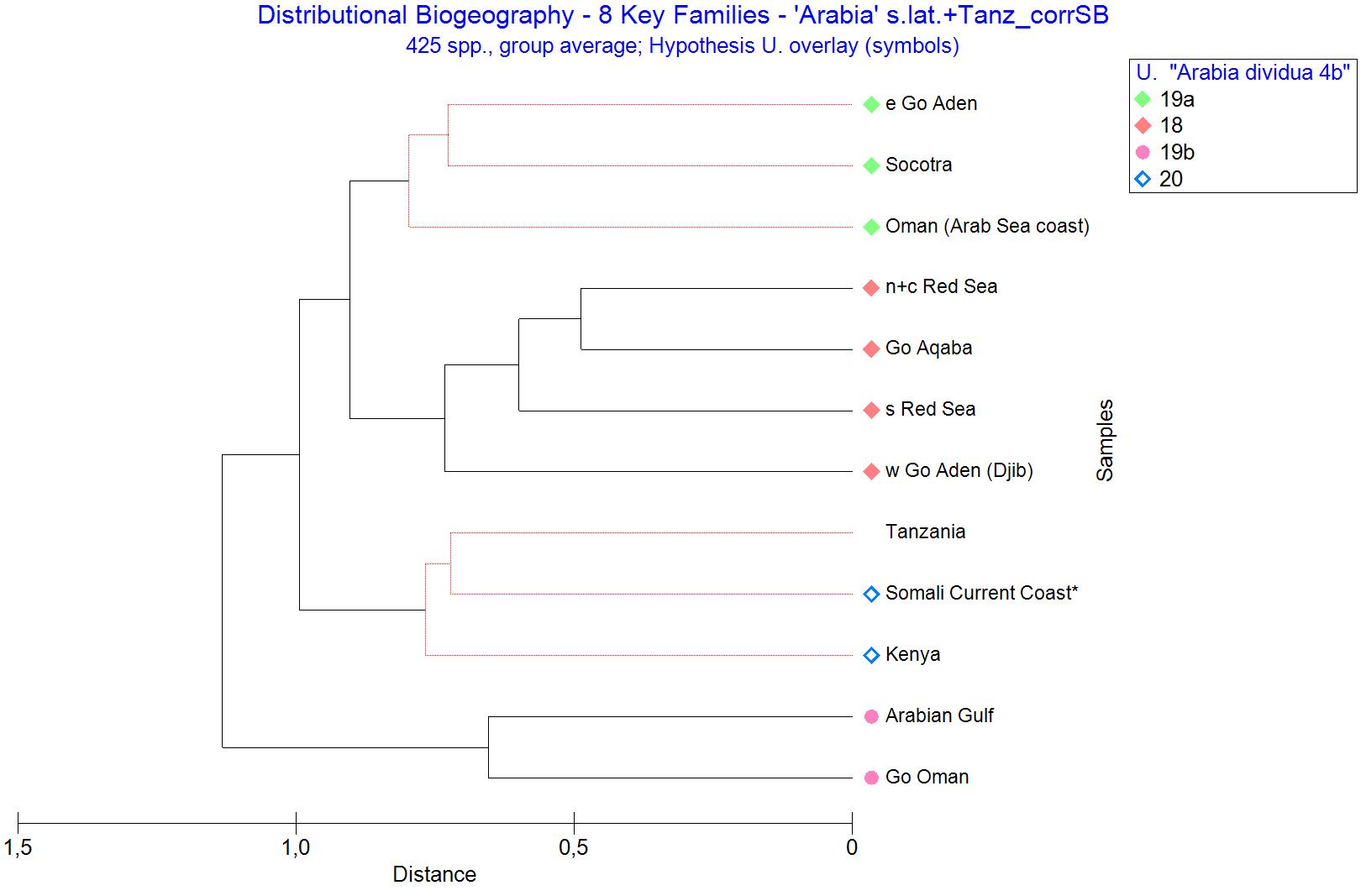


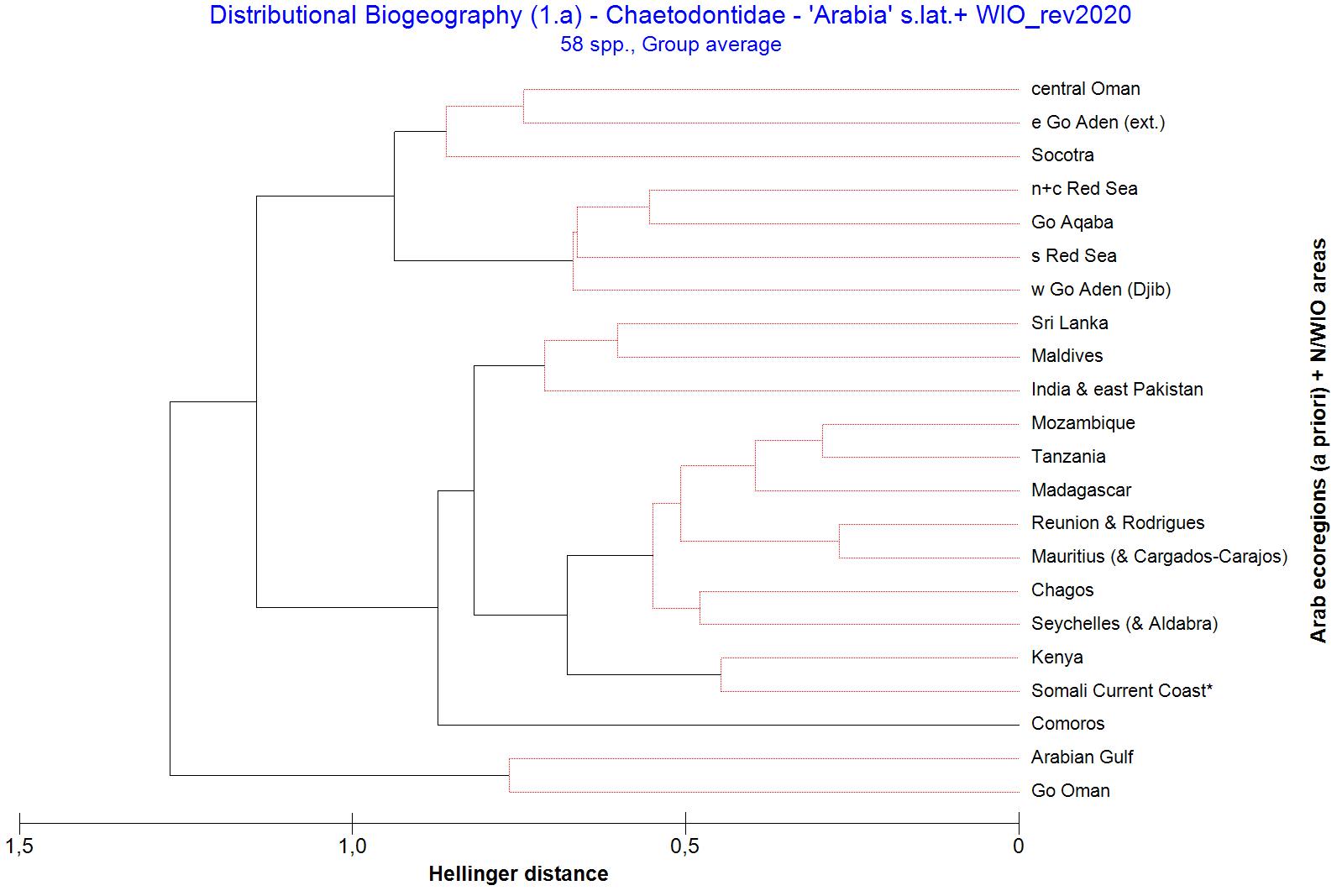


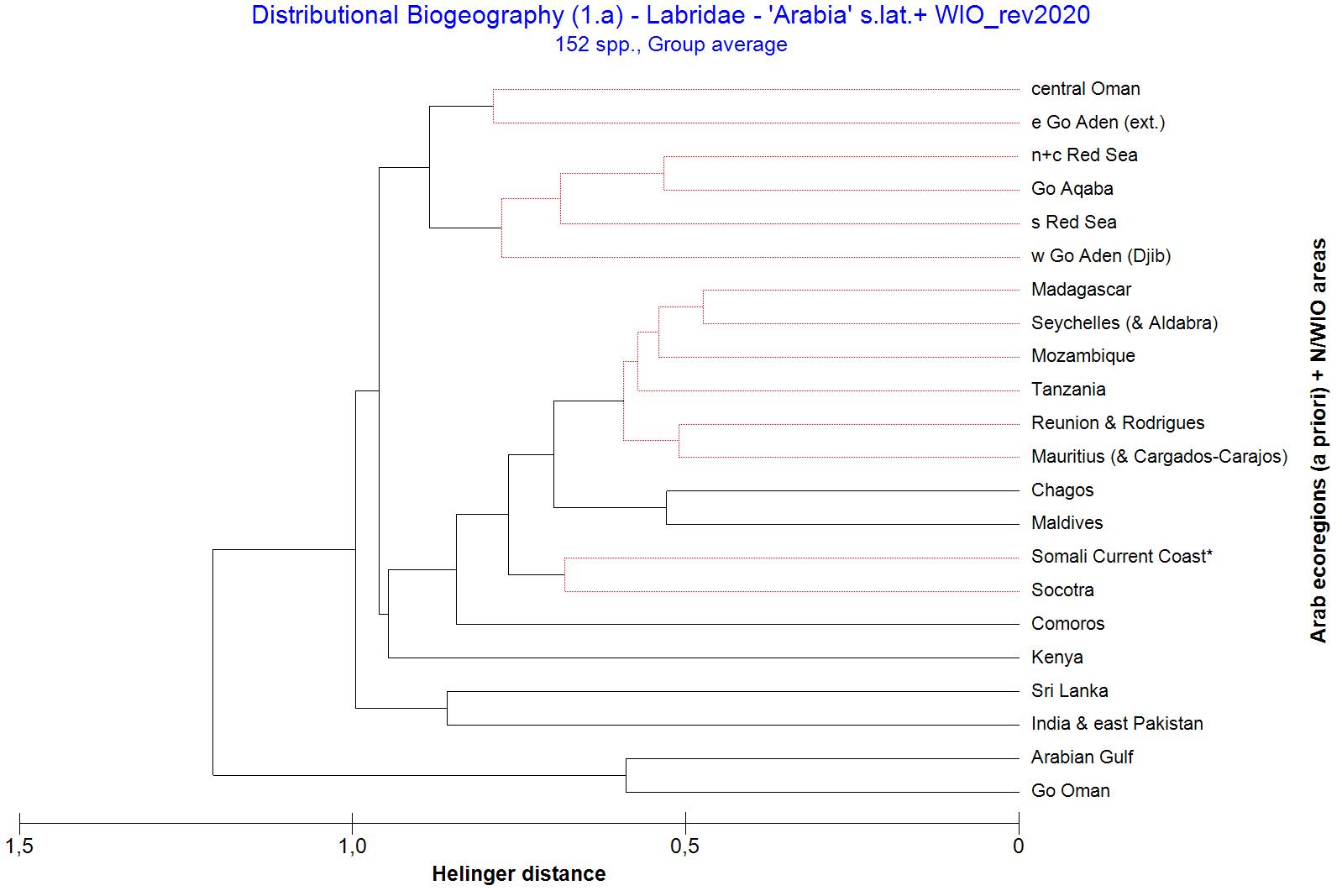


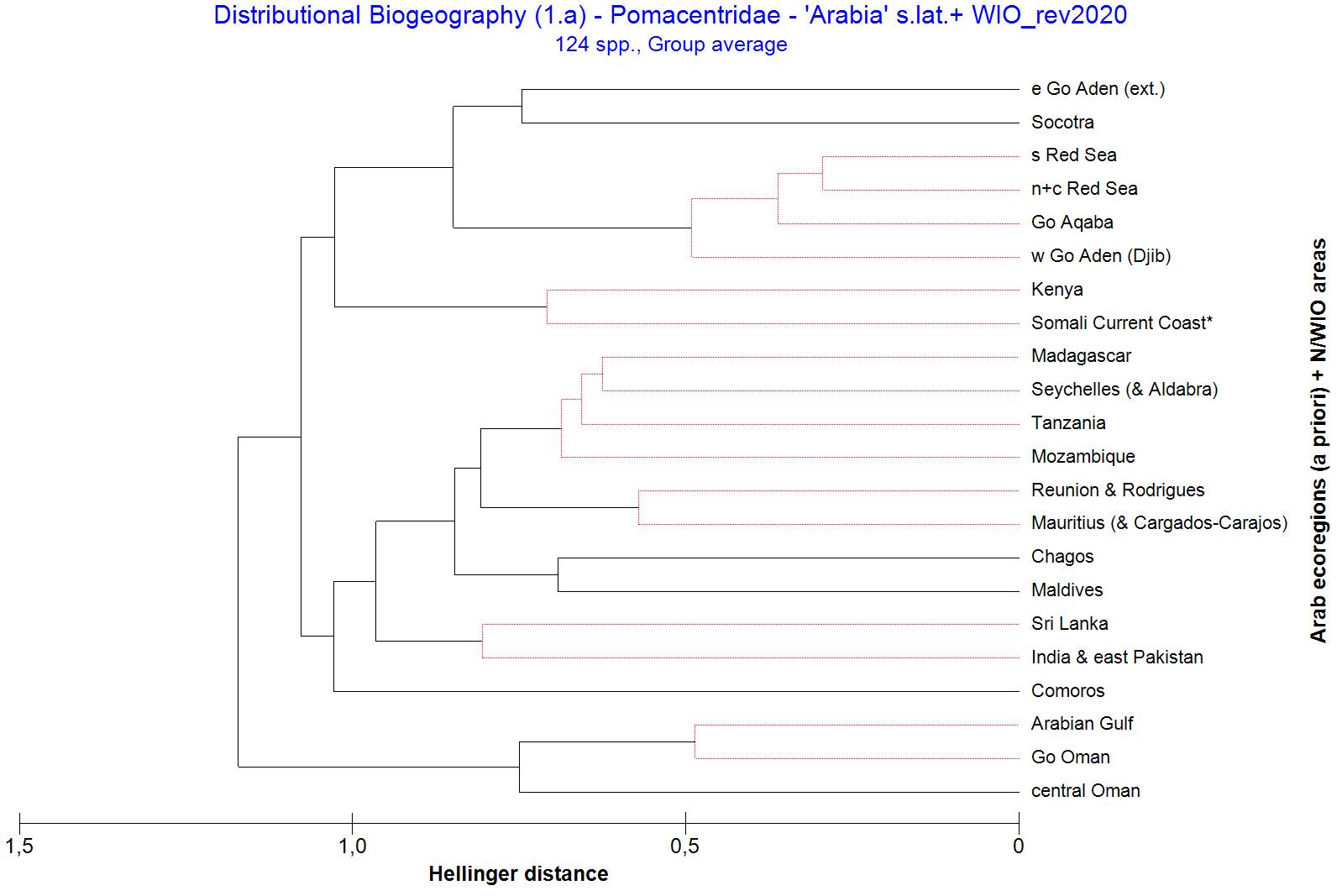


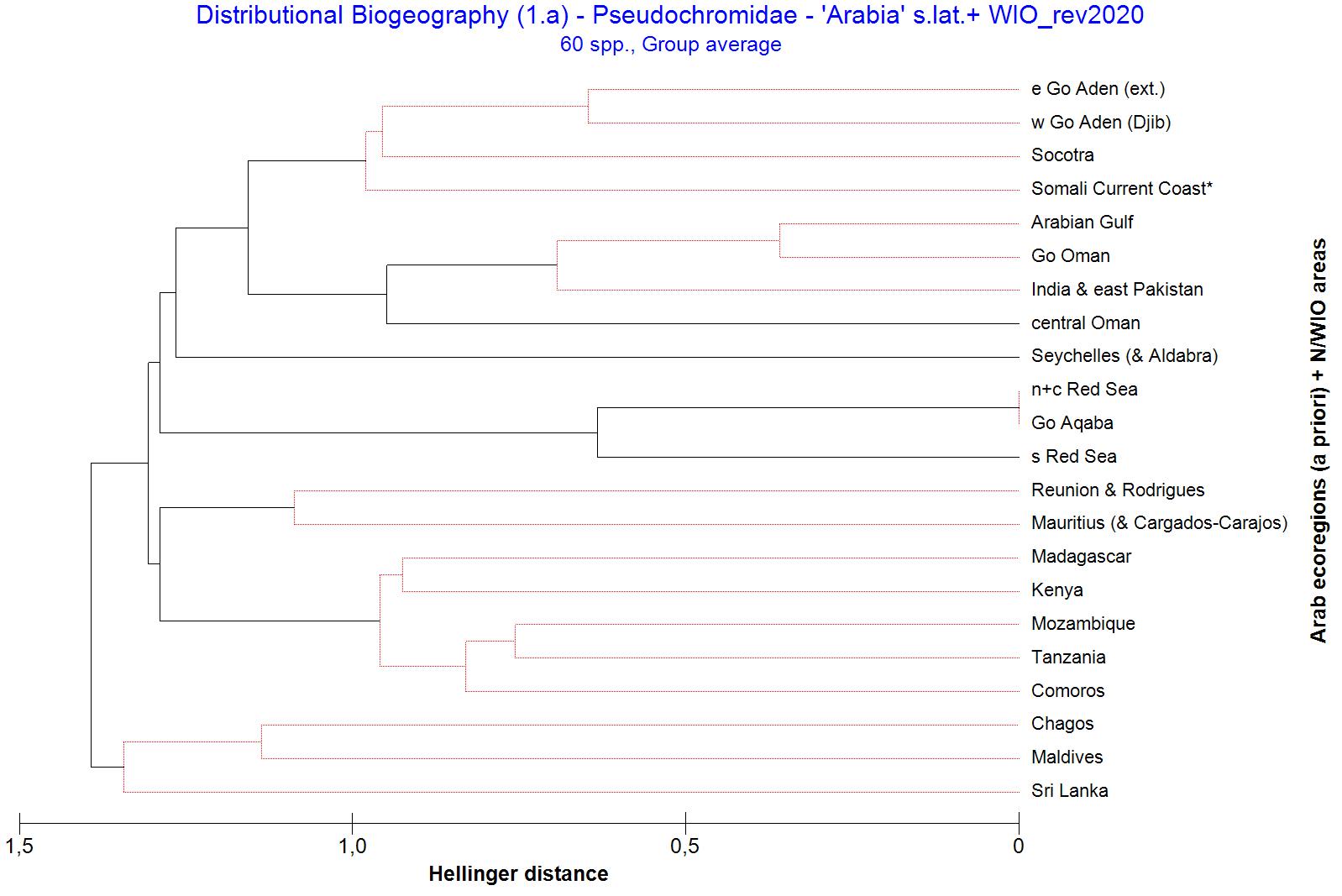

Supplement: S4 Fig — Supplementary figures and explorative analyses serving to validate results. (DOCX) [file pone.0267086.s008.docx]
